# Responses to sports tourist misbehavior: the influence of psychological distance, informal social control and personal implication-behavioral and EEG evidence

**DOI:** 10.3389/fpsyg.2026.1789417

**Published:** 2026-07-13

**Authors:** Yina Zhang, Jianlan Ding, Li Zhang, Haonan Shi, Yihang Huang, Zixuan Cheng, Wei Liu

**Affiliations:** 1 School of Sports Economics and Management, Xi’an Physical Education University, Xi’an, Shaanxi, China; 2 School of Physical Education, Shaanxi Normal University, Xi’an, Shaanxi, China; 3 School of Management, Xi’an Jiaotong University, Xi’an, Shaanxi, China

**Keywords:** behavioral experiment, ERP, informal social control, misbehavior intention, personal implication, psychological distance

## Abstract

**Introduction:**

With the rapid growth of sports tourism participation, the governance of tourist misbehavior has become a key issue in destination management. Based on Construal Level Theory (CLT), this study explores the behavioral and neural mechanisms through which psychological distance influences misbehavior intention.

**Methods:**

Guided by the Stimulus–Organism–Response (SOR) framework, this study constructed a moderated mediation model in which informal social control was introduced as a key mediator and personal implication as a moderator. The proposed model was tested through two online behavioral experiments and one electroencephalogram (EEG) experiment.

**Results:**

The two behavioral experiments showed that sports tourists exhibited higher misbehavior intention in non-local (vs. local) contexts, and this effect was partially mediated by informal social control. Moreover, personal implication exerted a moderated mediation effect, such that the indirect effect of psychological distance on misbehavior intention through informal social control was stronger in low personal implication contexts (environmental misbehavior) than in high personal implication contexts (public order misbehavior). The EEG experiment revealed that, during the early subconscious processing stage, local (vs. non-local) contexts elicited greater cognitive control (larger N200 amplitudes) for public order misbehavior but reduced cognitive control (smaller N200 amplitudes) for environmental misbehavior. During the later conscious processing stage, local (vs. non-local) contexts elicited greater attentional resource allocation (larger P300 amplitudes) for public order misbehavior. In addition, a significant negative correlation was observed between misbehavior intention and N200 amplitudes, indicating that heightened cognitive control inhibits misbehavior intention.

**Discussion:**

This study examines how psychological distance influences tourist misbehavior in sports tourism. Drawing on CLT, results show that non-local contexts increase misbehavior intention by weakening informal social control through an abstract mindset, whereas local contexts strengthen cognitive control and normative sensitivity, thereby reducing misbehavior. Informal social control serves as a key mediating mechanism, while personal implication moderates this relationship, indicating boundary conditions across different misbehavior types. Behavioral and ERP evidence jointly demonstrate that local contexts enhance cognitive control, reflected in stronger N200 responses, which suppress misbehavior intention. In contrast, non-local contexts reduce early conflict monitoring. These findings provide multi-level evidence linking psychological, and neural mechanisms in misbehavior decision-making, and provide practical guidance for destinations to curb tourists’ misbehavior.

## 1 Introduction

In recent years, large-scale sports events and sports tourism activities-such as marathons, international competitions, and outdoor events-have attracted a large number of participants. These activities are typically accompanied by clearly defined behavioral norms and regulatory frameworks designed to ensure smooth operation of events, enhance participant experience, and maintain the sustainability of the public environment. However, instances of tourists’ misbehavior have increasingly been reported, including queue-jumping, littering, landscape damage, and public urination, all of which disrupts event order, degrade environmental quality, and undermine the tourism image of destinations. Tourist misbehavior is defined as actions that violate widely accepted social norms and generate actual or potential harm to other individuals and/or the tourism environment. Existing research indicates that such misbehavior not only increases negative interactions among participants but also significantly diminish travel satisfaction and reduce revisit intention (Tsang et al., 2016). Within sports tourism contexts, individual misbehavior can disrupt the event progress, lower participant satisfaction, and erode the collective atmosphere and social experience of events (Tsaur et al., 2019). When accumulated over time, these behaviors may substantially damage the image of sports tourism destinations and hinder their long-term sustainable development (Campayo-Sanchez et al., 2026). Prior studies suggest that tourists are more likely to engage in misbehavior when they are away from their usual place of residences (McKercher et al., 2008; Bauer and McKercher, 2003). This observation raises an important research question: why are individuals more prone to engage in uncivilized or misbehavior when traveling outside their home environment?

The ethical decision-making model proposed by Jones (1991) identifies “proximity” as a critical factor influencing ethical judgments, encompassing dimensions of social, cultural, psychological, and physical closeness. Specifically, the closer a decision-maker is to a potential victim, the less likely they are to engage in misbehavior. This framework can provide a useful lens for explaining why participants in sports tourism context may be more prone to misbehavior when they are away from their usual place of residence. Prior research suggests that individuals are more likely to adhere to the norms of groups with which they feel closely connected, as such groups can more effectively exert informal social control over misbehavior (Nugier et al., 2009). Moreover, because individuals tend to avoid self-attribution of moral responsibility for negative consequences, when ethical issues are perceived as closely related to the self, they are more likely to form normatively appropriate behavioral intentions. Accordingly, when sports tourism participants travel away from home, their psychological closeness to the destination decreases, weakening perceived informal social control and increasing the propensity for misbehavior.

Existing research has explored misbehavior among various populations, including ordinary consumers (Tsang et al., 2016; Uriely et al., 2011; Xue et al., 2025; Sun et al., 2024; Jia et al., 2025; Meng et al., 2026), employees (Jones and Kavanagh, 1996; Vardi and Wiener, 1996; Bitner et al., 1994) and tourists, with a particular focus on restaurants (Knutson et al., 1999), hotels (Bitner et al., 1994; Harris and Reynolds, 2003), and airlines (Bitner et al., 1994). However, relatively few studies have specifically addressed misbehavior among sports tourism participants. Emerging evidence from media reports and short-form video platforms has increasingly highlighted the prevalence of such behaviors, including taking shortcuts, abruptly stopping during event to take photos, or engaging in other disruptive actions (Sun et al., 2024; Su et al., 2022; Volgger and Huang, 2019). Additional examples include deviating from designated routes, engaging in unsafe or inappropriate selfie-taking, and disregarding local cultural norms and traditions. Beyond disrupting public or event order, such behaviors also cause environmental degradation, including landscape damage, littering, and unauthorized carving. Such behaviors are widespread and persistent. By 2022, the U. S. National Park Service estimated that tourists’ improper behaviors over times had resulted in approximately USD 21.8 billion in maintenance and repair costs (Newsmedia, 2022). Despite substantial resources devoted to preventing such misbehavior, limited progress has been achieved in effectively curbing the occurrence of misbehavior.

Prior research has primarily examined the antecedents of misbehavior from the perspectives of social influence, individual characteristics, and situational factors (Su et al., 2022; Wan et al., 2021). Some studies highlight the roles of peer behavior (Sun et al., 2024), social norms (Su et al., 2022), and social identity (Wang et al., 2023). In addition, causal models of misbehavior suggest that such behavior arises from the interaction between individual traits and situational contexts, implying that its occurrence depends on both personal dispositions and environmental conditions (Jones and Kavanagh, 1996). Psychological distance has been shown to play a critical role in moral judgment and unethical decision-making (Zhan et al., 2020; Guo et al., 2019; Christensen and Gomila, 2012; Chen et al., 2017). For instance, it can influence pro-environmental behavior by shaping individuals’ mental construal of others, events, and consequences, as well as their perceived sense of responsibility (Thomsen, 2025).

However, relatively little research has systematically explained why individuals are more likely to rationalize or tolerate misbehavior when they are away from their usual place of residence (Wan et al., 2021). Given that sports tourism activities are highly situational, interactive, and norm-driven (Campayo-Sanchez et al., 2026; Gibson, 1998), this study draws on the SOR model proposed by Mehrabian and Russell (1974), which posits that external stimuli influence internal organismic states, thereby triggering behavioral responses. Building on this framework, the study develops and empirically tests a conceptual model of “psychological distance-informal social control-misbehavior intention” to examine how psychological distance shapes misbehavior in sports tourism contexts.

Existing studies indicate that psychological distance influences unethical judgment and behavioral decision-making by altering levels of abstract-concrete mental representations (Uriely et al., 2011; Zhan et al., 2020; Trope and Liberman, 2010; Trope et al., 2007). Employing two online experiments and one EEG experiment, this study provides robust and convergent empirical evidence that psychological distance influences misbehavior intention, with informal social control partially mediating this relationship. By differentiating public order misbehavior from environmental misbehavior according to the degree of personal implication involved, this study introduces personal implication as a key moderator. This approach is consistent with prior literature emphasizing the importance of behavioral visibility and self-relevance in shaping misbehavior intention (Tsaur et al., 2019; Sun et al., 2024; Wan et al., 2021; Chekroun, 2008). The findings suggest that the effect of psychological distance on perceptions of informal social control is contingent upon the extent to which the misbehavior directly involves individual self-interest and personal implication. By uncovering the pathway from psychological distance to misbehavior intention via informal social control, and by demonstrating how this mechanism varies across personal implication, this study extends the theoretical understanding of tourist misbehavior and its underlying psychological mechanisms. Furthermore, the results provide context-specific managerial implications for destinations, offering strategies to promote desirable tourist behavior and mitigate the negative consequences of misbehavior.

The remainder of this paper is organized as follows. Section 2 outlines the theoretical foundation and develops the hypotheses. Sections 3 and 4 present two behavioral experiments (study 1 and study 2) that sequentially test the proposed relationships, beginning with the effect of psychological distance on misbehavior intention and subsequently examining the mediating role of informal social control and the moderating role of personal implication. Section 5 employs an ERP experiment (study 3) providing neurophysiological evidence regarding the cognitive processes underlying misbehavior decisions. Finally, Section 6 discusses the theoretical implications, practical contributions, limitations, and future research directions.

## 2 Theoretical background and hypotheses

### 2.1 Sports tourism misbehavior

The manifestations of tourist misbehavior are diverse, including property damage, queue-jumping, littering, excessive food at buffets, fare evasion at attractions, disorderly conduct, and using flash photography in prohibited areas (Su et al., 2022; Volgger and Huang, 2019; Wan et al., 2021). Existing literature has conceptualized tourist misbehavior (Wang et al., 2023) and documented its negative consequences at both organizational and consumer levels. A variety of terms-such as deviant (Osgood et al., 1996), unethical (Schweitzer et al., 2004; Pan et al., 2020), aberrant (Fullerton and Punj, 1993), dysfunctional (Harris and Reynolds, 2003), problem (Bitner et al., 1994) and “Jaycustomers” (34)-have been employed to describe such behaviors, all of which converge on the notion of actions that violate widely accepted social norms during the consumption process. Building on this foundation, the study adopts the broader term “misbehavior in sports tourism,” defined as sports tourists’ failure or unwillingness to conform to generally accepted social norms, thereby disrupting the expected order of travel-related activities (Jia et al., 2025; Wan et al., 2021). Prior research has confirmed that such behaviors not only disrupt service order and interfere with routine operations (Meng et al., 2026), but also deteriorate the experiences of other tourists and undermine the overall image and reputation of the destination (Tsaur et al., 2019). For instance, during sports events and associated tourism activities, behaviors such as verbally abusing referees or staff, pushing or shoving others, damaging venue facilities, and littering can disrupt significantly disrupt event operations and service delivery processes. These actions increase management and monitoring costs for organizers and service providers, while also intensifying the emotional labor demands placed on frontline employees and volunteers, ultimately reducing service quality and job satisfaction (Harris and Reynolds, 2003; Grove and Fisk, 1997). Moreover, misbehavior often generates spillover effects through interpersonal interactions among tourists, thereby amplifying its negative consequences and further damaging destination image. Certain types of misbehavior can also lead to resource waste and environmental degradation (Juvan et al., 2018), and may even trigger resentment among local residents, ultimately constraining the sustainable development of tourism destinations (Shen et al., 2017).

Misbehavior has been extensively examined from the perspective of social influence (Wan et al., 2021), including factors such as social norms (Chien and Ritchie, 2018), social contagion (Su et al., 2022), and peer influence (Sun et al., 2024). Su et al. (2022) explored tourist misbehavior through a social influence framework and found that tourists were more likely to engage in inappropriate behavior after observing the uncivilized actions performed by others. In addition, other studies suggest that misbehavior may stem from cultural differences between tourists and destination, as well as unfamiliarity with local norms (Chien and Ritchie, 2018; Tolkach et al., 2017). Such cultural discrepancies often lead to unintentional violations of local accepted etiquette and behavioral standards (Jia et al., 2025; Wan et al., 2021). This tendency is further supported by psychodynamic sociological perspectives, which propose that the unfamiliarity and relative anonymity of destination may release suppressed misbehavior intention and immoral behaviors. Nevertheless, there remains a lack of systematic research examining the underlying psychological and behavioral mechanisms that drive tourist misbehavior.

### 2.2 Psychological distance and misbehavior intention

Psychological distance refers to “the emotional attachment and perceived connection that arises to another person” (Sun et al., 2024). According to CLT, the psychological distance between an individual and a target influences the individual’s mental representation, the way they construe related issues, and their motivation to engage in corrective action (Hamilton et al., 2021). Prior research indicates that individuals typically perceive greater closeness to in-group members than to out-group members (Wan et al., 2021; Trope et al., 2007; Fan et al., 2025). When tourists leave their usual place of residence, they often experience a sense of unfamiliarity and alienation, forming a psychological distance from the destination. Psychological distance is multidimensional, encompassing factors such as social distance and spatial distance (Trope and Liberman, 2010). Social distance refers to the perceived closeness to others based on identity, group affiliation, and value similarity (Akerlof, 1997). Spatial distance refers to the physical separation between an individual’s location or range of activity and a specific place or event (Cheng et al., 2024). In the context of sports tourism, local and non-local destinations differ along both of these dimensions. For spatial distance, local destinations are closely integrated with an individual’s daily environment, geographically near, and associated with low travel costs, making events there feel “close to me.” In contrast, non-local destinations are physically more distant, creating a sense of separation from daily life (Cheng et al., 2024). For social distance, local destinations typically evoke stronger identity and emotional attachment, leading individuals to view residents, public spaces, and social norms as part of “us,” thereby reducing perceived social distance (Wan et al., 2021; Magee and Smith, 2013). In comparison, people and norms in non-local destinations are more likely to be categorized as “they,” increasing social distance. Social and spatial distance make local destinations feel psychologically closer and non-local destinations more distant (Thomas and Tsai, 2012), providing a key basis for understanding tourists’ behavior across different contexts.

The factors underlying differences between local and non-local misbehavior. First, cultural differences with the destination and unfamiliarity with local social norms (Chien and Ritchie, 2018; Tolkach et al., 2017) may lead tourists to engage in non-local misbehavior. Second, psychodynamic and sociological studies suggest that tourists’ repressed aggression, deviant impulses, or self-indulgent needs are more likely to emerge when they are away from the local environment, increasing the likelihood of misbehavior (Uriely et al., 2011). In addition, the anonymity, situational isolation, and reduced sense of responsibility in non-local contexts diminish individuals’ awareness and self-restraint, further facilitating misbehavior. Notably, some scholars have highlighted that tourists temporarily detach themselves from the constraints of family and work environments, allowing them to set aside social norms and moral expectations that normally regulate their behavior (Wan et al., 2021; Turner and Ash, 1975). In other words, the sports tourism context may trigger a temporary shift in individuals’ “implicit values.” When individuals are local residents, social supervision and self-restraint often inhibit misbehavior. During travel, this restraint is weakened, making misbehavior more likely to occur. Scholars such as Eyal et al. (2008) and Fan et al. (2025) have introduced psychological distance to moral decision-making, showing that individuals are more likely to make altruistic choices for those with close relationships than for distant ones. Thomsen (2025) argues that psychological distance reduces the intensity of negative emotions, leading to stronger prosocial or morally guided behavior toward those with close psychological proximity than toward socially distant others. Therefore, the following hypothesis is proposed:

*H1*: Compared with the local context, participants in the non-local context will exhibit higher levels of misbehavior intention.

### 2.3 The mediating effect of informal social control

Both psychologists and sociologists have highlighted the critical role of social control in maintaining social norms and promoting ethical behavior (Wan et al., 2021; Thomsen, 2025; Wickes et al., 2017; Warner and Rountree, 1997). As sociologist Davis (1948) noted, “It is through them [social controls] that human society regulates the behavior of its members in such ways that they perform activities fulfilling societal needs” (p. 52). Individuals who violate social norms are subject to negative sanctions, which constitute a form of social control (Brauer and Chekroun, 2005). Research has shown that formal and informal social control are fundamentally different, yet both are essential mechanisms for restricting deviant behavior and criminal activities in society (Pate and Hamilton, 1992; Mumtaz, 2025). Unlike formal social systems, informal social control refers to sanctions imposed by others whose primary responsibility is not to ensure that group members comply with norms in a particular situation, whether verbal or non-verbal. Chekroun (2008) found that people often communicate their dissatisfaction with violators through angry glances, loud sighs, negative comments, or complaints. Collins and Frey (1992) showed that informal social control by peers is quite effective in preventing adolescents from driving under the influence of alcohol. The distinction between formal and informal social control can also be applied to sports tourism, which often involves interactions among participants sharing the same service environment (Pate and Hamilton, 1992). For example, stadium staff preventing tourists from using flash photography during a game represents formal social control, whereas other tourists expressing their disapproval of such violations through verbal or nonverbal cues exemplifies informal social control. Although formal social control is essential in modern society, the study focuses on informal social control for two main reasons. On the one hand, informal social control is often more effective than formal measures at curbing inappropriate behavior in shared environments (Wickes et al., 2017; Mumtaz, 2025). On the other hand, informal social control is more practical and feasible for managing behavior in sports tourism service settings (Wan et al., 2021).

In the context of sports tourism, informal social control plays a key mediating role between psychological distance and misbehavior intention. CLT suggests that when individuals perceive themselves as psychologically distant from the consequences of their actions or from those affected, their moral sensitivity and sense of responsibility for norm violations are attenuated, thereby increasing their likelihood of engaging in misbehavior intention (Trope and Liberman, 2010). Within this process, informal social control, defined as normative restraint spontaneously exercised by ordinary members of society through social interactions and evaluative feedback, directly shapes an individual’s judgments regarding the acceptability of behaviors. When psychological distance is close, individuals are more likely to anticipate potential social reactions, such as disapproving glances, negative evaluations, or verbal admonishments (Nugier et al., 2009; Warner and Rountree, 1997). Anticipation of these social sanctions heightens motivation to comply with norms, thereby reducing intentions to engage in misbehavior (Chekroun, 2008). Conversely, greater psychological distance leads individuals to view their actions as less relevant to others, diminish attention to potential social reactions, and perceive weaker informal social control, thereby lowering the psychological cost of norm violations and increasing misbehavior intentions (Wan et al., 2021; Wickes et al., 2017; Warner and Rountree, 1997). Furthermore, prior research has demonstrated that informal social control is often more immediate and effective than formal mechanisms in curbing deviance, particularly in highly interactive, public, and consumption-oriented contexts such as tourism and sports events (Wan et al., 2021; Akerlof, 1997; Brauer and Chekroun, 2005). Therefore, the following hypotheses are proposed:

*H2*: Informal social control mediates the relationship between psychological distance and misbehavior intention, such that participation in non-local (vs. local) contexts reduces perceived informal social control, thereby increasing individuals’ misbehavior intention.

### 2.4 The moderating effect of personal implication

In addition to psychological distance, personal implication has also been shown to further influence the degree to which individuals perceive informal social control. Personal implication refers to an individual’s subjective perception that a particular misbehavior or its consequences directly or indirectly affect their own interests (Chekroun, 2008; Brauer and Chekroun, 2005). Some forms of misbehavior exert direct negative effects on bystanders, whereas others do not. For instance, when an individual observes someone littering in their own yard versus littering in a stranger’s yard, the former situation evokes a stronger sense of personal implication, as the observer is a direct victim.

Whether an individual perceives “personal implication” directly influences their assessment of the social consequences of the behavior and their likelihood of enforcing social norms (Wan et al., 2021; Chekroun, 2008). Specifically, when tourists perceive their misbehavior poses a direct or potential threat to others in interests, experience quality, or group image-such as disrupting the viewing experience, creating safety hazards, damaging event reputation, or harming the destination’s image-they are more likely to perceive informal social control through verbal reminders, complaints, or informal exclusion (Warner and Rountree, 1997; Mumtaz, 2025). Conversely, when tourists perceive their misbehavior as unrelated to others interests, they experience a lower sense of personal implication. This reduced personal implication weakens their perception of informal social control, thereby increasing their inclination toward misbehavior. This sense of personal implication is closely related to what is often referred to as “egoism” (Chekroun, 2008; Ratner and Miller, 2001; Miller, 1999). Given that human motivation is largely driven by self-interest (Holmes et al., 2002), individuals are more likely to feel affected by events that bear direct relevance to themselves than by those that do not.

Sports tourism is often characterized by a high level of publicity, interactivity, emotional engagement, and group identification (Campayo-Sanchez et al., 2026; Gibson, 1998). Even in the absence of direct victimization, the collective identity and normative commitment embedded in sports tourism settings may still activate bystanders’ subjective sense of personal implication. Personal implication may increase as a function of the extent to which the observer feels victimized (Wan et al., 2021; Chekroun, 2008). Public order misbehavior, including queue jumping, quarrelling, pushing, seat occupying, and disrupting the viewing order, directly interferes with tourists’ experiences and often threatens perceived fairness, comfort, and safety. As such, it is more likely to elicit stronger feelings of personal involvement (Wan et al., 2021; Thomas and Tsai, 2012; Wickes et al., 2017). In contrast, environmental misbehavior, such as littering, damaging public facilities, and trampling on grass primarily affects the environment and public space. Although these behaviors undermine public interests and destination image, their consequences are often indirect, delayed, and less personally salient to individual tourists (Osgood et al., 1996; Brauer and Chekroun, 2005). Drawing on simulation theory, which posits that individuals infer the consequences of others’ actions by imagining themselves in a given situation. When misbehavior generates more direct and negative consequences for other tourists within the same service environment, misbehavior actors are more likely to anticipate social disapproval and intervention from others. Accordingly, personal implication is expected to function as a boundary condition in the relationship between psychological distance and perceived informal social control. Specifically, higher levels of personal implication should weaken the negative impact of psychological distance on perceived informal social control, exhibit stronger misbehavior intentions. Therefore, the following hypotheses are proposed:

*H3a*: Personal implication plays a moderating effect between psychological distance and informal social control. That is, the negative effect of psychological distance on perceived informal social control is weaker in public order misbehavior (vs environmental misbehavior) contexts.

*H3b*: Personal implication plays a moderated mediating effect between psychological distance and misbehavior intention. That is, psychological distance exerts a weaker negative effect on perceived informal social control in the context of public order misbehavior (vs environmental misbehavior), inhibiting misbehavior intention.

### 2.5 ERPs as a measure of cognition and attention

As an important research tool in cognitive neuroscience, EEG enables the capture of the dynamic process of brain information processing with millisecond-level temporal resolution, uncovering the neural mechanisms underlying individuals’ cognitive and emotional responses (Cahn and Polich, 2006; Cohen, 2017). In recent years, EEG techniques have been gradually introduced into tourism management research and have demonstrated distinct value across a range of topics, including destination marketing and brand perception (Bastiaansen et al., 2018), tourism experience and emotional responses (Li et al., 2022), virtual tourism and immersive experiences (Zhang and Xiao, 2025), as well as ethical judgment and decision-making (Lee et al., 2017). By enabling real-time monitoring of brain activity, EEG allows researchers to objectively assess individuals’ implicit cognitive processing and allocation of attentional resources in tourism contexts. This approach helps mitigate the subjective bias associated with self-reported measures, which are commonly present in traditional questionnaire and interview methods, thereby enhancing the explanatory depth and methodological rigor of tourism behavior research (Venkatraman et al., 2015).

In the field of ethical decision-making, EEG has become an important tool for examining individuals’ cognitive and emotional responses to different marketing stimuli (Pozharliev et al., 2022). ERP research by Zhan, Xiao (Zhan et al., 2020) demonstrated that, compared with decisions involving strangers, decisions involving friends elicit stronger negative emotions and greater cognitive load in unethical decision-making contexts, reflecting a tendency toward “self-interested altruism.” These effects are manifested in ERP components such as the N200, P300, and LPP components. Building on this line of research, the present study employs ERP analysis to further explore differences in tourists’ cognitive processing and attentional resource allocation when they are exposed to different combinations of psychological distance and personal implication. Specifically, this approach enables a fine-grained examination of the temporal dynamics underlying moral evaluation and behavioral intention formation in sports tourism contexts.

In neuromarketing research, the N200 and P300 components have been widely recognized as two of the most extensively studied ERPs (Pozharliev et al., 2015). The N200 is a negative-going component that typically peaks between 250 and 350 ms following stimulus onset and is primarily distributed over the frontal brain regions (Li et al., 2022; Folstein and Van Petten, 2008). This component reflects early cognitive control processes involved in detecting and suppressing conflicting information, and is associated with automatic, rapid, and largely subconscious response mechanisms (Bartholow et al., 2005; Grützmann et al., 2022).

According to CLT, local contexts are psychologically closer to individuals at both the spatial and social levels, thereby making misbehavior more strongly associated with self-relevance and social identity (Trope and Liberman, 2010; Trope et al., 2007). Public order misbehavior (e.g., queue jumping, arguing, pushing) is typically characterized by a high level of personal implication (Zhan et al., 2020; Chekroun, 2008; Ratner and Miller, 2001). When such behavior occurs in a local context, individuals are more likely to perceive it as a direct threat to the self, thereby activating stronger processes of norm violation detection and conflict monitoring. According to conflict monitoring theory, when individuals detect violation of social norms and anticipate the need for behavioral regulation, activity in the anterior cingulate cortex (ACC) is enhanced, which is reflected in an increased N200 amplitude (Folstein and Van Petten, 2008; Botvinick et al., 2001). Therefore, compared with non-local contexts, local public order misbehavior is more likely to enhanced N200 amplitudes, reflecting stronger automatic cognitive control process involved in detecting and evaluating norm conflicts, supporting H4a. In contrast, environmental misbehavior (e.g., littering, landscape damage) typically does not elicit immediate interpersonal conflict and is associated with lower levels of personal implication and weaker conflict salience (Sun et al., 2024; Ratner and Miller, 2001). As a result, such behaviors are less likely to activate early-stage conflict monitoring mechanisms, leading to comparatively attenuated N200 responses. In local contexts, individuals are more attentive to the concrete consequences of the behavior, which enhances its self-relevance. When individuals become aware of the negative consequences and attribute responsibility to themselves, internal norms are activated (Schwartz, 1977), thereby increasing sensitivity to norm violations and eliciting normative responses. N200 is widely regarded as a key indicator of conflict monitoring and norm violation detection (Folstein and Van Petten, 2008). In local contexts, although environmental misbehavior is generally low in personal implication, the heightened self-relevance increases the likelihood of cognitive conflict, thereby eliciting larger N200 amplitudes and indicating greater cognitive control for detecting and evaluating norm violations (Fan et al., 2025; Yao et al., 2025). By contrast, in non-local contexts, increased psychological distance attenuates attention to concrete consequences and reduces the self-relevance of the behavior. Individuals are more likely to externalize responsibility, thereby inhibiting the norm activation process, reducing conflict perception and resulting in smaller N200 amplitudes, thereby supporting H4b. Therefore, the following hypotheses are proposed.

*H4*: Compared with the non-local context, participants in the local context will exhibit higher levels of cognitive control (larger N200 amplitudes).

*H4a*: For public order misbehavior, participants in the local (vs. non-local) context will exhibit higher levels of cognitive control (larger N200 amplitudes).

*H4b*: For environmental misbehavior, participants in the local (vs. non-local) context will exhibit higher levels of cognitive control (larger N200 amplitudes).

The P300 is a positive-going ERP component that typically emerges approximately 250-600 ms following stimulus onset and is primarily distributed over the central-parietal regions (Nieuwenhuis et al., 2005). During later stages of attentional processing, the P300 is generally interpreted as reflecting controlled, resource-dependent cognitive processing, particularly in relation to stimulus evaluation, moral judgment, and norm-based reasoning in complex social contexts (Meidenbauer et al., 2018).

**Figure 1:**
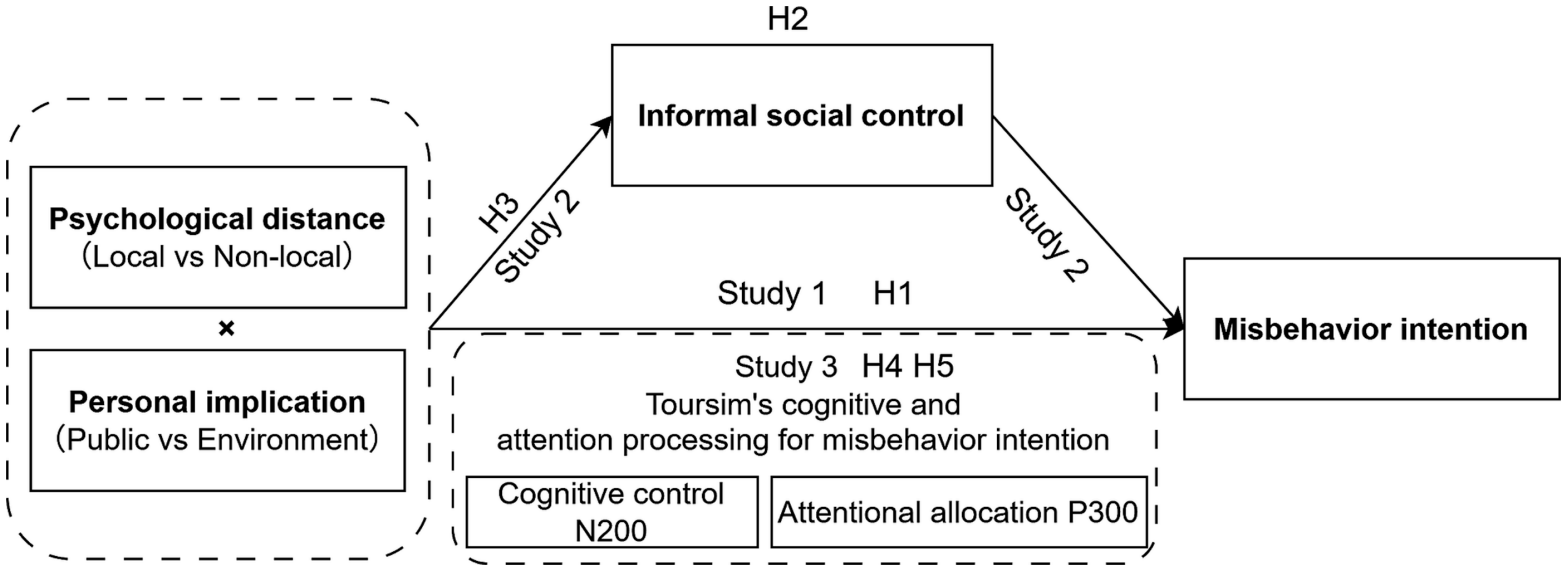
Conceptual framework.

Public order misbehavior in local contexts can be conceptualized as highly concrete and self-relevant stimuli, which increases individuals’ attentional and emotional engagement (Liberman and Chaiken, 1996). The CLT posits that when psychological distance is close, situations are represented in a more concrete and vivid manner. This representational fit facilitates attentional arousal and processing, thereby promoting deeper processing of norm-related information and resulting in enhanced P300 amplitudes. Specifically, in cases of public order misbehavior, local identity activates individuals’ sense of responsibility and heightens expectations of potential interpersonal conflict in shared order, leading to greater allocation of attentional and emotional resources to evaluation of norm violations (Greene et al., 2001). This intensified processing is reflected at the neural level as an increased P300 response. In contrast, environmental misbehavior (e.g., littering) is typically characterized by lower self-relevance and reduced immediacy of consequences. Its outcomes are often associated with weaker emotional arousal and lower temporal urgency, resulting in reduced cognitive and emotional engagement. Consequently, such behaviors tend to elicit attenuated P300 amplitudes. Therefore, the following hypotheses are proposed (Figure 1).

*H5*: Compared with the non-local context, participants in the local context will exhibit higher levels of attentional engagement (larger P300 amplitudes).

*H5a*: For public order misbehavior, participants in the local (vs. non-local) context will exhibit higher levels of attentional engagement (larger P300 amplitudes).

*H5b*: For environmental misbehavior, participants in the local (vs. non-local) context will exhibit higher levels of attentional engagement (larger P300 amplitudes).

## 3 Study 1

In study 1, through one online experiment, we test H1 using a 2 × 2 between-subjects design, with psychological distance (local vs. non-local) and personal implication (public order misbehavior vs. environment misbehavior) as the factors.

### 3.1 Pretest

According to the conceptualization of psychological distance, Xi’an (local) was selected as the stimulus representing low psychological distance. To ensure comparability and contextual equivalence between destinations, Nanjing (non-local) was selected as the stimulus representing high psychological distance destination. Subsequently, a total of 60 participants were recruited (age range 18–60, M_age_ = 27.97 years, male proportion 46.43%). Using a 7-point Likert scale adapted from previous studies (Trope and Liberman, 2010; Bar-Anan et al., 2006), participants were asked to evaluate the perceived levels of spatial and social distance associated with Xi’an and Nanjing. As shown in Table 1, the psychological distance manipulation was effective [M_xia*n =*_ 3.033, SE = 0.206, M_nanjing_ = 5.175, SE = 0.171; *t* (30) = −8.007, *p <* 0.001].

**Table 1:** Independent-samples *t*-test results for psychological distance.

Variables	Psychological distance (Mean ± SD)		*t*	*p*	BC 95% confidence interval (CI)	
	Xian (*n =* 30)	Nanjing (*n =* 30)			Lower	Upper
Spatial distance	3.117 ± 1.291	5.200 ± 1.157	−6.583	0.001	−2.717	−1.450
Social distance	2.950 ± 1.028	5.150 ± 1.421	−6.869	0.001	−2.841	−1.559
Psychological distance	3.033 ± 1.123	5.175 ± 0.936	−8.007	0.001	−2.677	−1.606

To develop experimental scenarios that effectively enhance participants’ sense of involvement, the study first identified common forms of inappropriate behavior in sport tourism context. To this end, a pilot study was conducted using offline data collection (*n =* 48, 18 males and 30 females, M_age_ = 21.9) to explore representative instances of misbehavior in sport tourism settings.

To evaluate these behaviors, a focus group discussion involving 10 participants was conducted at a university in Xi’an. All participants had tourism experience within the past 5 years (M_age_ = 23). They were asked to describe frequently observed instances of misbehavior during sport tourism activities. Based on their responses, four typical forms of misbehavior were identified: queue-jumping, littering, environmental damage, and disruptive behaviors that interfere with event order.

Participants were then asked to complete an online questionnaire and rate the degree of misbehavior using a 7-point Likert scale. One-sample *t*-test results showed that participants viewed all four actions as misbehavior: queue-jumping [M = 5.927, *t* (47) = 37.128, *p <* 0.001], disrupting order [M = 5.792, *t* (47) = 39.949, *p <* 0.001], littering [M = 5.604, *t* (47) = 36.570, *p <* 0.001], and landscape damage [M = 6.083, *t* (47) = 42.297, *p <* 0.001] (Table 2).

**Table 2:** Result of the *t*-test on the degree of misbehavior.

Misbehavior types	Mean	SD	*t*	*P*	BC 95% CI	
					Lower	Upper
Queue-jumping	5.927	1.106	37.128	0.001	5.606	6.248
Disrupting order	5.792	1.004	39.949	0.001	5.500	6.083
Littering	5.604	1.062	36.570	0.001	5.296	5.913
Landscape damage	6.083	0.996	42.297	0.001	5.794	6.373

### 3.2 Design and procedure

In this study, participants were recruited and financially compensated through Credamo, a professional online research platform in China. This approach facilitates the inclusion of respondents from diverse demographic backgrounds and enhances the external validity of the findings. Participants were required to be at least 18 years old and to have prior experience in sports tourism. Participants were randomly assigned to four experimental groups. To ensure data quality, attention check items were embedded (e.g., “If you are reading this page, please select option 5”), and respondents who failed these checks were excluded. After removing incomplete or inattentive responses, a total of 312 valid responses were retained from 320 completed questionnaires (age range: 18–58 years, M_age_ = 32.99, 47.81% male).

#### 3.2.1 Stimulus

They were asked to imagine themselves attending a major sporting event in either Xi’an or Nanjing. In the public order misbehavior condition, participants were instructed to imagine that, at a critical moment in the game, a spectator seated in the front row suddenly stood up and raised his arms to cheer for the team. In the environmental misbehavior condition, participants were asked to imagine that, after the game, while attempting to exit the stadium quickly, they observed several spectators cutting across the lawn outside the venue.

#### 3.2.2 Measures

After reading the assigned scenario, participants completed a questionnaire measuring psychological distance, misbehavior intention, misbehavior degree, personal implication, and demographic characteristic. All items were rated on a 7-point Likert scale. Psychological distance (*α* = 0.971) comprised social distance and spatial distance (Liberman and Chaiken, 1996; Bar-Anan et al., 2006). Misbehavior degree (*α* = 0.919) was measured across three dimensions: sports tourism environment, sports tourism resource, and sports tourism order (Xue et al., 2025; Sun et al., 2024; Wan et al., 2021). The measurement of personal implication was adapted from (Brauer and Chekroun, 2005) (“To what extent do you think other tourists would personally suffer the consequences of your standing up to watch the game or cutting across the lawn”; 1 = very unlikely, 7 = very likely). The measurement of misbehavior intention was adapted from Schaefers et al. (2016) (i.e., “how likely is it that you would cutting lines?”; 1 = very unlikely, 7 = very likely). Demographic variables included gender, age, education level, and sports involvement.

### 3.3 Results

#### 3.3.1 Measurement model

The overall Kaiser-Meyer-Olkin (KMO) value for the data is 0.862, indicating that the correlation structure is adequate for factor analysis. Bartlett’s test of sphericity is highly significant, *χ*^2^ (45) = 4276.025, *p <* 0.001, suggesting that the dataset is suitable for underlying factor extraction and structure identification.

To ensure the quality of the measurement model, confirmatory assessment of reliability, convergent validity, and discriminant validity were conducted. Reliability and convergent validity were evaluated using average variance extracted (AVE) and composite reliability (CR), with recommended threshold values of 0.50 and 0.70, respectively. As shown in Table 3, AVE values ranged from 0.708 to 0.894, while CR values ranged from 0.922 to 0.971. All indicator exceeded the recommended thresholds, indicating strong internal consistency and satisfactory convergent validity across all constructs, thereby confirming the overall measurement quality of the model.

**Table 3:** Item loadings and reliability.

Variables	Indicator	Standardized loading	AVE	CR	Cronbach’s a
Psychological distance	D1	0.950	0.894	0.971	0.971
	D2	0.928			
	D3	0.962			
	D4	0.942			
Misbehavior degree	U1	0.961	0.798	0.922	0.919
	U2	0.915			
	U3	0.795			

Convergent and discriminant validity were further examined using standardized factor loadings and the square root of the AVE (see Table 3). All items exhibited standardized loadings above 0.70, indicating satisfactory indicator reliability and supporting adequate convergent validity. Discriminant validity was assessed using the Fornell-Larcker criterion, which requires that the square root of the AVE for each construct exceed its correlations with other constructs (Fornell and Larcker, 1981). As shown in Table 4, the square root of the AVE values for psychological distance (0.946), and misbehavior degree (0.893) was all higher than their corresponding inter-construct correlations (see Table 4). These results collectively provide strong support for the discriminant validity of the constructs employed in this study.

**Table 4:** Validity and correlations for constructs.

Variables	1	2
Psychological distance	**0.946**	
Misbehavior degree	0.229	**0.893**

Values in bold on the diagonal indicate the square root of the AVE.

#### 3.3.2 Manipulation check

Psychological distance. A one-way analysis of variance showed that there are significant differences between local and non-local in both spatial distance and social distance dimensions [Spatial distance: M_local_ = 3.522, SE = 0.143, M_non-local_ = 5.388, SE = 0.082; *F* (1,312) = 103.209, *p <* 0.001; Social distance: M_local_ = 3.282, SE = 0.126, M_non-local_ = 5.240, SE = 0.071; *F* (1,312) = 104.859, *p <* 0.001] (Table 5).

**Table 5:** Results for psychological distance.

Variables	Psychological distance (Mean ± SD)		*F*	*p*
	Local (*n =* 156)	Non-local (*n =* 156)		
Spatial distance	3.522 ± 1.783	5.388 ± 1.023	103.209	0.001
Social distance	3.282 ± 1.572	5.240 ± 0.886	104.859	0.001
Psychological distance	3.410 ± 1.664	5.319 ± 0.920	120.764	0.001

*p < 0.05, **p < 0.01.

Misbehavior degree. A One-way analysis of variance showed that there was a significant difference in the degree of misbehavior between public order misbehavior and environmental misbehavior [tourism environment: M_public_ = 4.460, SE = 0.134, M_environment_ = 5.130, SE = 0.130; *F* (1,312) = 18.315, *p <* 0.001, $\eta_{p}^{2}\;$ = 0.056, tourism resources: M_public_ = 4.150, SE = 0.113, M_environment_ = 4.340, SE = 0.073; *F* (1,312) = 2.077, *p* = 1.151, $\eta_{p}^{2}\;$ = 0.007, tourism order: M_public_ = 5.180, SE = 0.122; M_environment_ = 4.628, SE = 0.087; *F* (1,312) = 13.674, *p <* 0.001, $\eta_{p}^{2}\;$ = 0.042] (Table 6).

**Table 6:** Results for misbehavior degree.

Items	Misbehavior degree (Mean ± SD)		*F*	*p*
	Public order (*n =* 156)	Environment (*n =* 156)		
Sport tourism environmental	4.460 ± 1.355	5.130 ± 1.422	18.315	0.001
Sport tourism resources	4.150 ± 1.040	4.340 ± 1.303	2.077	0.151
Sport tourism order	5.180 ± 1.426	4.628 ± 1.198	13.674	0.001

*p < 0.05, **p < 0.01.

Personal implication. The result indicated a significant difference in perceived personal implication between public order misbehavior and environmental misbehavior [M_public_ = 5.250, SE = 0.125, M_environment_ = 4.571, SE = 0.114; *F* (1,312) = 16.145, *p <* 0.001]. The participants believed that their misbehavior would result in greater suffering for other tourists in the public order misbehavior (high personal implication) than in the environmental misbehavior (low personal implication) condition (Table 7).

**Table 7:** Results for personal implication.

Variables	Personal implication (Mean ± SD)		*F*	*p*
	Public order misbehavior (*n =* 156)	Environmental misbehavior (*n =* 156)		
Personal implication	5.250 ± 1.564	4.571 ± 1.419	16.145	0.001

#### 3.3.3 Results

To test H1, PROCESS Macro Model 1 was employed (Hayes and Rockwood, 2017). The results indicated a significant direct effect of psychological distance on misbehavior intention (*β* = 0.433, SE = 0.110, 95% CI = [0.216, 0.650] (see Table 8), Therefore, H1 was supported.

**Table 8:** Testing the effect of psychological distance on misbehavior intention.

Variables	Misbehavior intention				BC 95% CI	
	*β*	SE	*t*	*p*	Lower	Upper
Constant	−0.150	0.514	−0.292	0.770	−1.162	0.862
Psychological distance	0.433	0.110	3.930	0.001	0.216	0.650
Public order misbehavior	0.635	0.056	10.188	0.001	0.461	0.683
Environmental misbehavior	0.811	0.041	17.191	0.001	0.629	0.793

For public order misbehavior, psychological distance significantly increased tourists’ misbehavior intention (*β* = 0.635, SE = 0.023, 95% CI = [0.461, 0.683]). For environmental misbehavior, psychological distance also exerted a significant positive effect on misbehavior intention (*β* = 0.811, SE = 0.041, 95% CI = [0.629, 0.793]).

## 4 Study 2

Study 2 further examined the effects of psychological distance and personal implication on misbehavior intention, with perceived informal social control introduced as a mediating variable. The study employed a 2 × 2 experimental design, in which psychological distance (local vs. non-local) and personal implication (public order misbehavior vs. environmental misbehavior) were treated as a between-subjects factors.

### 4.1 Design and procedure

In this study, participants were recruited from Credamo and financially compensated, following the same screening criteria and attention checks as in Study 1. Study 2 also employed a diverse online sample to enhance the generalizable of the analyses. Of the initial 340 respondents, 320 valid responses (Age range = 18–60 years, M_age_ = 27.98, 42.80% male) were retained for analysis. Participants were randomly assigned to one of four experimental conditions, with 80 individuals in each group. Study 2 followed a procedure similar to that of Study 1.

#### 4.1.1 Stimulus

Participants were instructed to imagine themselves attending a major sporting event in either Xi’an or Nanjing. In the public order misbehavior condition, they imagined waiting in a long and slowly moving queue to collect their marathon race package. An opening beside the queue offered an opportunity to cut in line by pretending to join a friend positioned further ahead, thereby shortening the waiting time. In the environmental misbehavior condition, participants imagined running a marathon and holding an empty water bottle after finishing the drink. Because waste bins along the course were relatively far apart, disposing of the bottle on the ground would allow them to maintain their running pace without interruption.

#### 4.1.2 Measures

After reading the assigned scenario, participants completed a questionnaire measuring psychological distance, misbehavior intention, personal implication. Same as study 1, all items were rated on a 7-point Likert scale. Perceived informal social control was measured using the scale developed by Sampson et al. (1997), which consists of four dimensions: supervision, willingness to intervene, norm enforcement and social support. Psychological distance and personal implication were operationalized in the same manner as in Study 1.

### 4.2 Results

#### 4.2.1 Measurement model

The overall KMO value was 0.823, indicating that the correlation pattern was adequate for adequate factor extraction. Bartlett’s test of sphericity was significant, *χ*^2^ (105) = 3466.109, *p <* 0.001, suggesting that the correlation matrix significantly differed from the identity matrix and was therefore suitable for factor analysis.

The set of measures used in Study 2 replicated those in Study 1, with the addition of perceived informal social control as a construct. The reliability and validity indicators for psychological distance met the recommended standards (see Tables 9, 10). For perceived informal social control (Sampson et al., 1997), the AVE was 0.660 and the CR was 0.907. All standardized factor loadings exceeded 0.70 (Table 9). The square root of its AVE (0.813) exceeded its correlations with other constructs (Table 10), thereby satisfying the Fornell-Larcker criterion.

**Table 9:** Item loading and reliability.

Variables	Indicator	Standardized loading	AVE	CR	Cronbach’s a
Psychological distance	D1	0.948	0.734	0.916	0.921
	D2	0.935			
	D3	0.740			
	D4	0.785			
Informal social control	I1	0.801	0.660	0.907	0.906
	I2	0.857			
	I3	0.829			
	I4	0.786			

**Table 10:** Pearson correlations and discriminant validity.

Variables	1	2
Psychological distance	**0.857**	
Informal social control	−0.325	**0.813**

Values in bold on the diagonal indicate the square root of the AVE.

#### 4.2.2 Manipulation check

Psychological distance. A one-way analysis of variance showed that there were significant differences in spatial distance and social distance between local and non-local [Spatial distance: M_local_ = 3.572, SE = 0.124, M_non-local_ = 5.222, SE = 0.093; *F* (1,320) = 113.766, *p <* 0.001, $\eta_{p}^{2}\;$ = 0.333; Social distance: M_local_ = 3.056, SE = 0.094, M_non-local_ = 4.700, SE = 0.103; *F* (1,320) = 138.556, *p <* 0.001, $\eta_{p}^{2}\;$ = 0.405] (Table 11).

**Table 11:** Results for psychological distance.

Variables	Psychological distance (Mean ± SD)		*F*	*p*
	Local (*n =* 160)	Non-local (*n =* 160)		
Spatial distance	3.572 ± 1.563	5.222 ± 1.177	113.766	0.001
Social distance	3.056 ± 1.191	4.700 ± 1.305	138.556	0.001
Psychological distance	3.198 ± 1.257	5.077 ± 0.923	232.081	0.001

*p < 0.05, **p < 0.01.

Personal implication. An independent-samples *t*-test revealed a significant difference in personal implication between public order misbehavior and environmental misbehavior [M_public_ = 5.125, SE = 0.095, M_environment_ = 4.363, SE = 0.106; *F* (1,320) = 6.903, *p <* 0.01] (Table 12).

**Table 12:** Results for personal implication.

Variables	Personal implication (Mean ± SD)		*F*	*p*
	Public order misbehavior (*n =* 160)	Environment misbehavior (*n =* 160)		
Personal implication	5.125 ± 1.201	4.363 ± 1.339	6.903	0.009

#### 4.2.3 Moderated mediation analysis

Moderation and moderated mediation analyses were conducted using Hayes’s PROCESS macro (Models 1 and 7) (Hayes and Rockwood, 2017), with 5,000 bootstrap resamples employed to test the hypotheses.

##### 4.2.3.1 Testing of the mediation effect

The direct effect results are summarized in Table 13. The BC 95% CI of psychological distance on informal social control was significant (*β* = −0.603, SE = 0.144, *p <* 0.001, 95% CI = [−0.887, −0.319]). Additionally, informal social control on misbehavior intention was also significant (*β* = −0.282, SE = 0.076, *p <* 0.001, 95% CI = [−0.436, −0.136]). The direct effect of psychological distance on misbehavior intention was also significant (*β* = 0.413, SE = 0.202, *p* = 0.042, 95% CI = [0.015, 0.811]).

**Table 13:** Results of informal social control as a mediator.

Mediation analysis	Pathway	*β*	SE	*t*	*p*	BC 95% CI	
						Lower	Upper
Mediator model	PD → ISC	−0.603	0.144	−4.179	0.001	−0.887	−0.319
Outcome model	ISC → MI	−0.282	0.076	−3.753	0.002	−0.436	−0.136
	PD → MI	0.413	0.202	2.040	0.042	0.015	0.811

Indirect effect analysis further showed that informal social control significantly mediated the relationship between psychological distance and misbehavior intention (*β* = 0.082, SE = 0.023, 95% CI = [0.027, 0.118]; see Table 14). Therefore, H2 was supported. The results indicate that informal social control as a mediator reduced the direct effect from 0.413 to 0.260 (*β* = 0.260, SE = 0.067, *p <* 0.001, 95% CI = [0.129, 0.392]), demonstrating a partial mediation effect.

**Table 14:** Bootstrap results of indirect effect.

Effect	*β*	SE	*t*	*p*	BC 95% CI	
					Lower	Upper
Total effect	0.342	0.065	5.295	0.001	0.215	0.469
Direct effect: PD → MI	0.260	0.067	3.889	0.001	0.129	0.392
Indirect effect: PD → ISC → MI	0.082	0.023	4.410	0.001	0.027	0.118

##### 4.2.3.2 Testing of moderated effect

**Figure 2:**
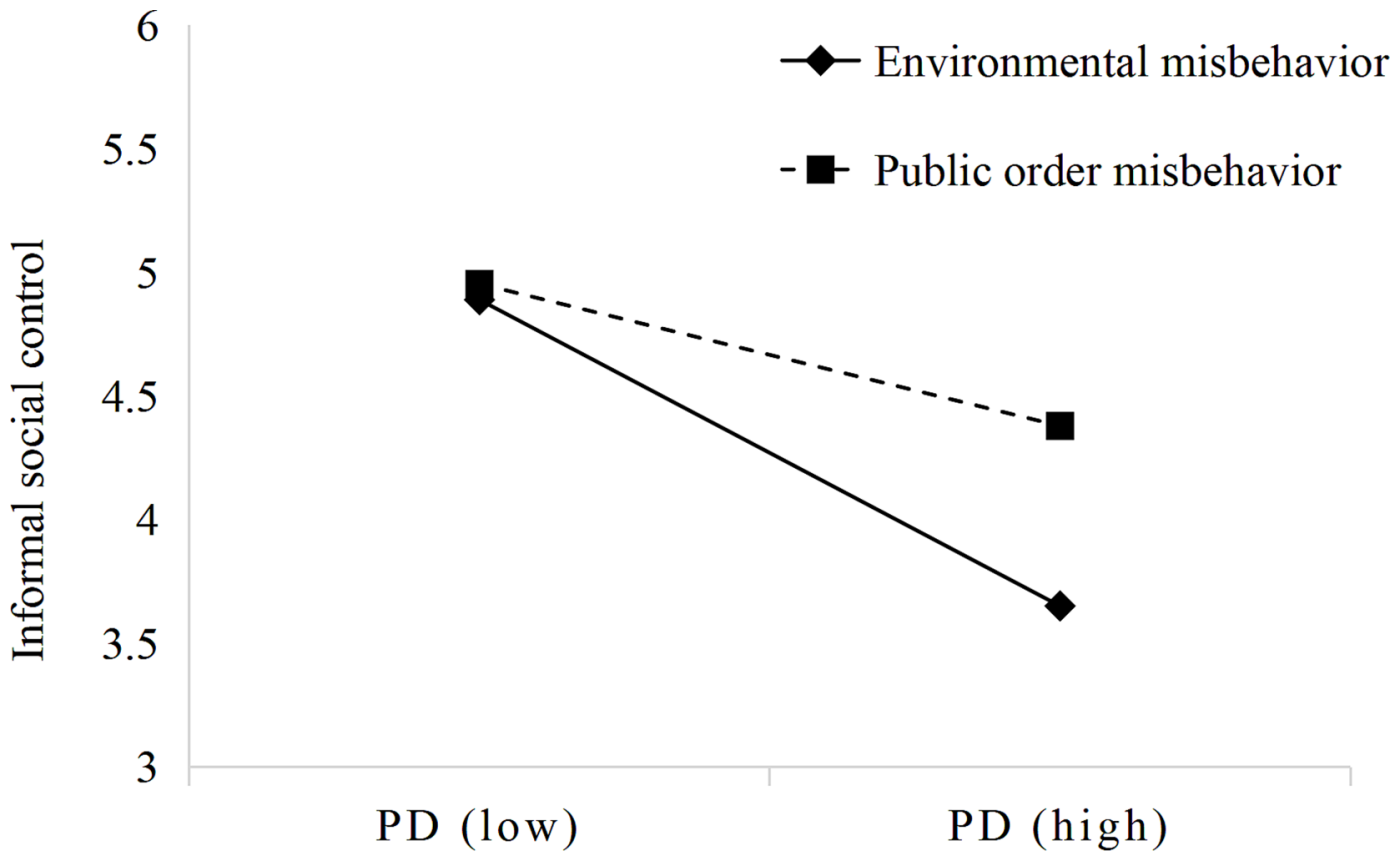
Simple slope analysis of the moderating effect of personal implication.

The results revealed a significant positive moderating effect of personal implication on the relationship between psychological distance and perceived informal social control (Moderation index = 0.229, SE = 0.099, 95% CI = [0.034, 0.425]), providing support for H3a. Simple slope analyses indicated that when personal implication was low, increases in psychological distance significantly reduced individuals’ perceptions of informal social control. However, when personal implication was high, this negative effect was substantially attenuated. In other words, compared with environmental misbehavior (low personal implication), the decline in perceived informal social control associated with greater psychological distance was less pronounced in public order misbehavior (high personal implication). Overall, the results indicate that personal implication buffers the negative impact of psychological distance on perceived informal social control (Table 15; Figure 2).

**Table 15:** Results of moderated effect of personal implication.

Pathway	*β*	SE	*t*	*p*	BC 95% CI	
					Lower	Upper
PD*PI→ISC	0.229	0.099	2.479	0.021	0.034	0.425
Public order misbehavior	−0.199	0.077	−2.589	0.010	−0.349	−0.048
Environmental misbehavior	−0.428	0.063	−6.792	0.001	−0.552	−0.304

Public order misbehavior represents high level of personal implication; environmental misbehavior represents low level of personal implication.

##### 4.2.3.3 Testing of moderated mediation effect

**Figure 3:**
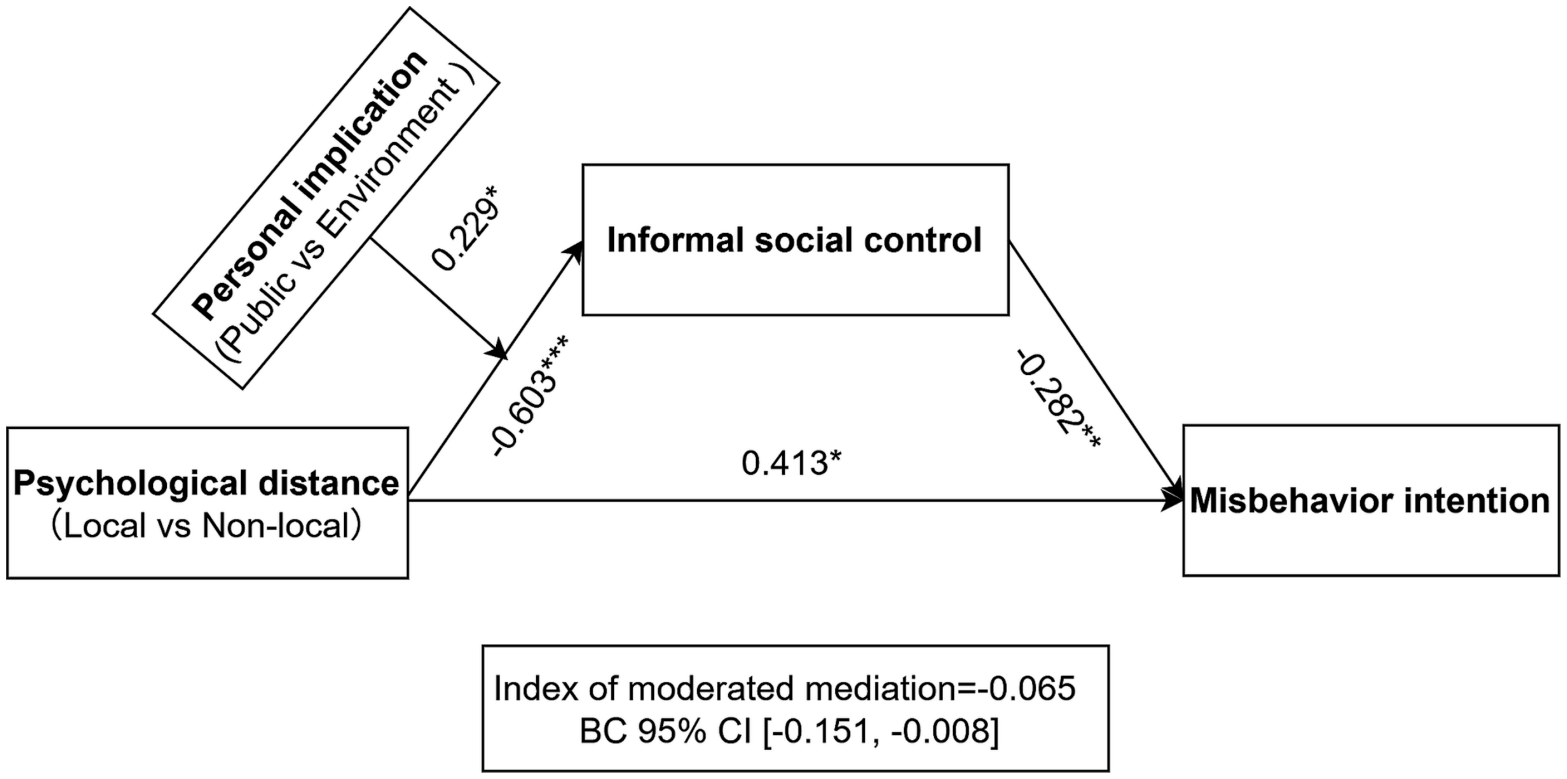
Personal implication serves as a moderator in the mediation of informal social control between psychological distance on misbehavior intention. *p < 0.05, **p < 0.01, ***p < 0.001.

The results further confirmed a significant moderated mediation model, in which informal social control mediated the effect of psychological distance on misbehavior intention, and that this mediating effect varied across personal implication (moderated mediation index = −0.065, SE = 0.037, 95% CI = [−0.151, −0.008]), H3b was supported. Specifically, for public order misbehavior, the conditional indirect effect of psychological distance on misbehavior intention was significant (*β* = 0.057, SE = 0.028, 95% CI = [0.009, 0.123]). For environmental misbehavior, the conditional indirect effect was also significant (*β* = 0.122, SE = 0.045, 95% CI = [0.048, 0.221], see Table 16). Specifically, the indirect effect of psychological distance on misbehavior intention via informal social control was stronger in low personal implication contexts. However, in high personal implication contexts, the strength of this indirect effect diminished significantly. The path estimates and their significance are shown in Figure 3.

**Table 16:** Conditional effect of psychological distance on misbehavior intention at values of the moderator for study 2.

Pathways	Moderator: Personal implication	Effect	SE	BC 95% CI	
				Lower	Upper
	Moderated mediation index	−0.065	0.037	−0.151	−0.008
PD → ISC → MI	Public order misbehavior	0.057	0.028	0.009	0.123
	Environmental misbehavior	0.122	0.045	0.048	0.221

Public order misbehavior represents high level of personal implication; environmental misbehavior represents low level of personal implication.

## 5 Study 3

### 5.1 Design and procedure

Two online experiments (Studies 1–2) indicate that psychological distance increases individuals’ misbehavior intentions, and that this relationship is partially mediated by perceived informal social control. Furthermore, the indirect effect was contingent upon the level of personal implication. These behavioral findings are consistent with the predictions of construal level theory and moral decision-making theory. Despite these insights, few studies have integrated online behavioral experiments with neurophysiological measures to capture the temporal dynamics underlying such decision-making processes. To address this gap, we conducted an ERP experiment (Study 3) that simultaneously manipulated psychological distance (local vs. non-local) and personal implication (public order misbehavior vs. environmental misbehavior). This design enabled us to examine not only behavioral outcomes but also the underlying dynamics of cognitive control and attentional resource allocation, thereby linking observable misbehavior intentions to their neural temporal processes.

#### 5.1.1 Subject

Based on previous studies (Guo et al., 2019; Thomsen, 2025; McTeague et al., 2011), the required sample size was estimated using G*Power 3.1.9. Assuming an effect size of *f* = 0.25, an alpha level of *α* = 0.05, and a desired statistical power of 0.90, the *a priori* power analysis indicated that a minimum total sample size is 36, with 18 participants allocated to each group. Participants received course credit for their participation. A total of 48 university students were initially recruited, of whom 8 were excluded due to extreme response patterns. The final sample consisted of 40 participants (17 males; M_age_ = 22.65 ± 2.55 years). All participants had normal or corrected-to-normal vision, no color blindness or severe color vision deficiency, were right-handed, reported no history of mental illness, and had not participated in similar experiments previously. The study was approved by the Ethics Committee of Xi’an Physical Education University, and written informed consent was obtained from all participants.

#### 5.1.2 Stimulus and procedure

##### 5.1.2.1 Experimental protocol

A two-factor mixed experimental design was employed: 2 (psychological distance: local vs. non-local) × 2 (personal implication: public order vs. environment). Psychological distance served as the between-subjects factor, whereas personal implication was treated as the within-subjects factor. The dependent variables included misbehavior intention, as well as the N200 and P300 components of ERPs.

In this study, two sports tourism destinations-local (Xi’an) and non-local (Nanjing)-were used as stimuli to manipulate psychological distance. In addition, established scales were employed to assess participants’ perceived spatial distance and social distance between local and non-local destinations (Trope and Liberman, 2010; Bar-Anan et al., 2006). In the moral decision-making task, psychological distance was operationalized by presentation destinations names (i.e., Xi’an and Nanjing), followed by images depicting misbehavior. All images were standardized in size, clearly labeled by misbehavior type, and annotated with signs indicating that such behaviors were prohibited.

This experimental design was adapted from prior EEG studies on moral decision-making (Guo et al., 2019), in which similar paradigms have been successfully applied. In line with established EEG experimental design and prior research (Zhan et al., 2020; Grützmann et al., 2022; Greene et al., 2001; Egner and Hirsch, 2005), a stimulus-decision paradigm was employed to examine automatic cognitive responses by analyzing how initial stimuli influence subsequent decision-making (Meidenbauer et al., 2018). Using this approach, EEG techniques were applied to investigate how the interaction between psychological distance and personal implication affects cognitive control and the allocation of cognitive resources during misbehavior decision-making.

##### 5.1.2.2 Stimuli

In EEG research, averaging across multiple trials is essential for improving the signal-to-noise ratio (SNR), as recorded EEG signals comprise both task-related neural activity and noise arising from ongoing brain processes and environmental sources (Luck, 2014). Across repeated trials, random noise tends to cancel out, whereas stimulus-locked neural responses remain stable. Therefore, tens to hundreds of trials are typically required to obtain reliable ERPs (Odom et al., 2010). Based on this principle, a repeated-measures design with a sufficient number of trials was adopted. Participants completed 80 trials in each experimental condition, resulting in a total of 160 trials (80 trials × 2 conditions). The experiment included 40 unique conditions (2 personal implication × 4 misbehavior subcategories × 5 stimulus images). The misbehavior subcategories were operationalized as ecologically valid behavioral scenarios embedded in sports tourism contexts (The scenario examples are provided in the Supplementary material). Specifically, public order misbehavior included queue jumping, crossing the track for photo-taking, unauthorized packing of event supplies, and expressing dissatisfaction through booing. Environmental misbehavior comprised littering, spitting in public spaces, graffiti, and vegetation destruction. All stimulus materials were developed based on realistic sports tourism scenarios and were carefully matched across subcategories in terms of visual complexity, background setting, actor presence, and scene salience to minimize potential confounding effects.

Each stimulus condition was presented twice, yielding 80 valid trials. Similar repeated presentation designs have been widely employed in neuromarketing and social cognition research (Telpaz et al., 2015). In each trial, participants were randomly presented with an image depicting misbehavior along with a brief textual description. Specifically, the misbehavior images were drawn from four subcategories identified in the pretest, with five images selected for each subcategory to maintain stimulus diversity and reduce participant fatigue.

##### 5.1.2.3 Procedure

**Figure 4:**
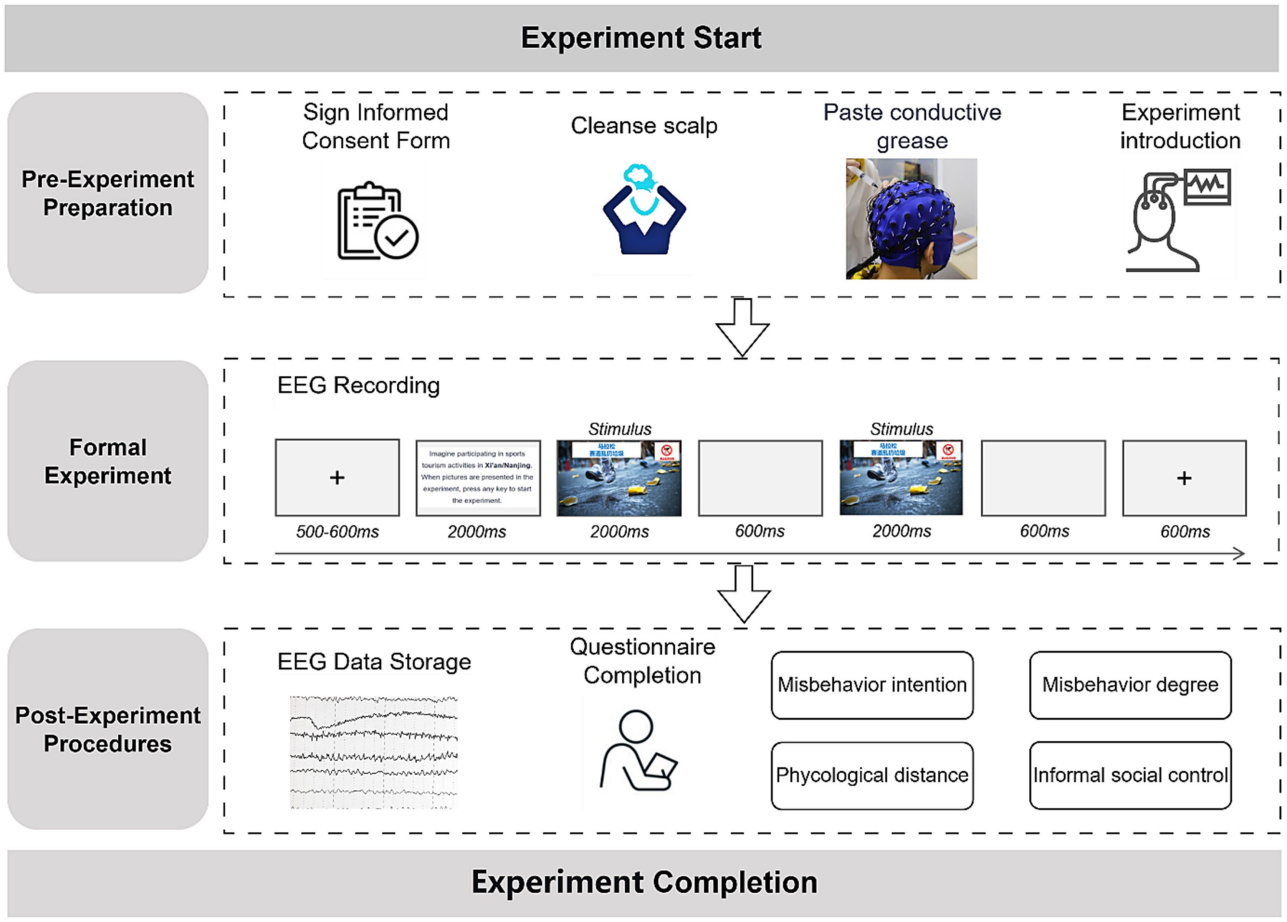
The procedure of the experiment in study 3.

The experimental procedure consisted of three stages: pre-experimental preparation, the EEG task, and a post-experimental questionnaire (see Figure 4). Prior to the experiment, all participants provided written informed consent and received a detailed explanation of the EEG procedures and equipment. To ensure data quality, participants were instructed to cleanse their scalp before electrode placement. Conductive gel was applied to the electrodes to maintain impedance below 5kΩ. After completing a brief practice session to become familiar with the procedure, participants proceeded to the formal experiment. Stimuli were presented using E-Prime 3.0 software, and a prime-probe paradigm was employed. Each trial began with a fixation cross displayed for 600 ms, followed by an image depicting misbehavior in a sports tourism context, which was presented for 2000 ms. To minimize extended deliberation and capture immediate responses, participants were instructed to indicate misbehavior intention while the image was displayed. Responses were recorded via the keyboard, with the F key indicating “no misbehavior intention” and the J key indicating “misbehavior intention.” After completing the EEG task, participants completed the same questionnaire as in Study 1 to assess perceived psychological distance, misbehavior degree, personal implication, and misbehavior intention.

### 5.2 EEG data recording, pre-processing, and data analysis

#### 5.2.1 EEG data recording

EEG data were recorded using a 64-channel Neuroscan EEG system, with signal acquisition and processing conducted via Curry 8 software. Electrodes were positioned in accordance with the international 10–20 system. EEG signals were recorded with a band-pass filter of 0.1–100 Hz and a sampling rate of 500 Hz. The ground electrode was positioned anterior to Fz, and the reference electrode was placed at FCz. Throughout the experiment, electrode impedance was maintained below 5 kΩ to ensure high-quality data acquisition.

#### 5.2.2 EEG data pre-processing

EEG data were analyzed using MATLAB (R2024b; The MathWorks, Inc., Natick, MA). First, raw EEG data were subjected channel localization, and invalid or noisy electrodes were removed. Ocular artifacts, including horizontal and vertical eye movement, were corrected using a regression-based procedure (Semlitsch et al., 1986). The data were then digitally band-pass filtered from 0.1 to 30 Hz with a 24 dB/octave roll-off to attenuate low-frequency drift and high-frequency noise (Luck, 2014). Secondly, the data were visually inspected, and segments containing excessive noise or movement artifacts were rejected. Bad channels were interpolated using spherical spline interpolation, and the data were subsequently re-referenced to the average of bilateral mastoid electrodes (M1 and M2). Thirdly, independent component analysis (ICA) was performed to identify and remove residual artifacts related to eye movements and muscle activity. Components were flagged for rejection using a minimum probability threshold of 90%. Rejection decisions were further validated through combined visual inspection of component topographies, time courses, and power spectra. Specifically, components exhibiting frontal-dominant scalp distributions characteristic of ocular artifacts, transient time-course patterns, elevated low-frequency (0–3 Hz) power, or high correlation with electrooculogram (EOG) signals (r > 0.30) were marked for removal. This procedure ensured the removal of physiological artifacts while preserving neural signal integrity. Finally, ERP waveforms were averaged for each participant across the four experimental conditions: local public order misbehavior, local environmental misbehavior, non-local public order misbehavior, and non-local environmental misbehavior.

#### 5.2.3 EEG data analysis

The N200 component was analyzed within a 170–300 ms time window using 6 electrodes (F3, Fz, F4, FC3, FCz, FC4) located in the frontal and frontal-central scalp regions, following ERP guidelines outlined by Picton et al. (2000) and prior research on N200 (Folstein and Van Petten, 2008; Jin et al., 2024; Kang et al., 2018). A repeated-measures ANOVA with a 2 (psychological distance: local vs. non-local) × 2 (personal implication: public order misbehavior vs. environmental misbehavior) × 6 (electrodes) design was conducted to examine the effects of psychological distance and personal implication on N200 average amplitude across electrodes. This analytic approach is consistent previous EEG studies in marketing and consumer neuroscience, which treat electrodes sites as a within-subject factor to capture neural responses across scalp regions (Yao et al., 2025).

For the P300 component, the analysis focused on a 250–380 ms time window using 6 electrodes (P3, Pz, P4, PO3, POz, PO4) located in the parietal and parieto-occipital regions of the scalp. Electrode selection followed established ERP methodological guidelines (Picton et al., 2000; Lu et al., 2019) and prior research on P300 (Pozharliev et al., 2015; Kropotov et al., 2024). A comparable repeated-measures ANOVA was conducted to assess the effects of psychological distance and personal implication on P300 average amplitude at these electrode sites. In addition, Pearson correlation analyses were performed to examine the relationships between misbehavior intention and the amplitudes of both the N200 and P300 components.

### 5.3 Results

#### 5.3.1 Behavior results

1. *Misbehavior intention*. A repeated measures ANOVA with a 2 (psychological distance) × 2 (personal implication) design revealed that the main effect of psychological distance on misbehavior intention was significant [*F* (1,40) = 15.134, *p <* 0.001, $\eta_{p}^{2}\;$ = 0.285]. The personal implication did not have a significant main effect [*F* (1,40) = 0.113, *p* = 0.739, $\eta_{p}^{2}\;$ = 0.003]. However, the interaction effect was found to be significant [*F* (1,40) = 4.281, *p <* 0.05, $\eta_{p}^{2}\;$ = 0.101].

**Figure 5:**
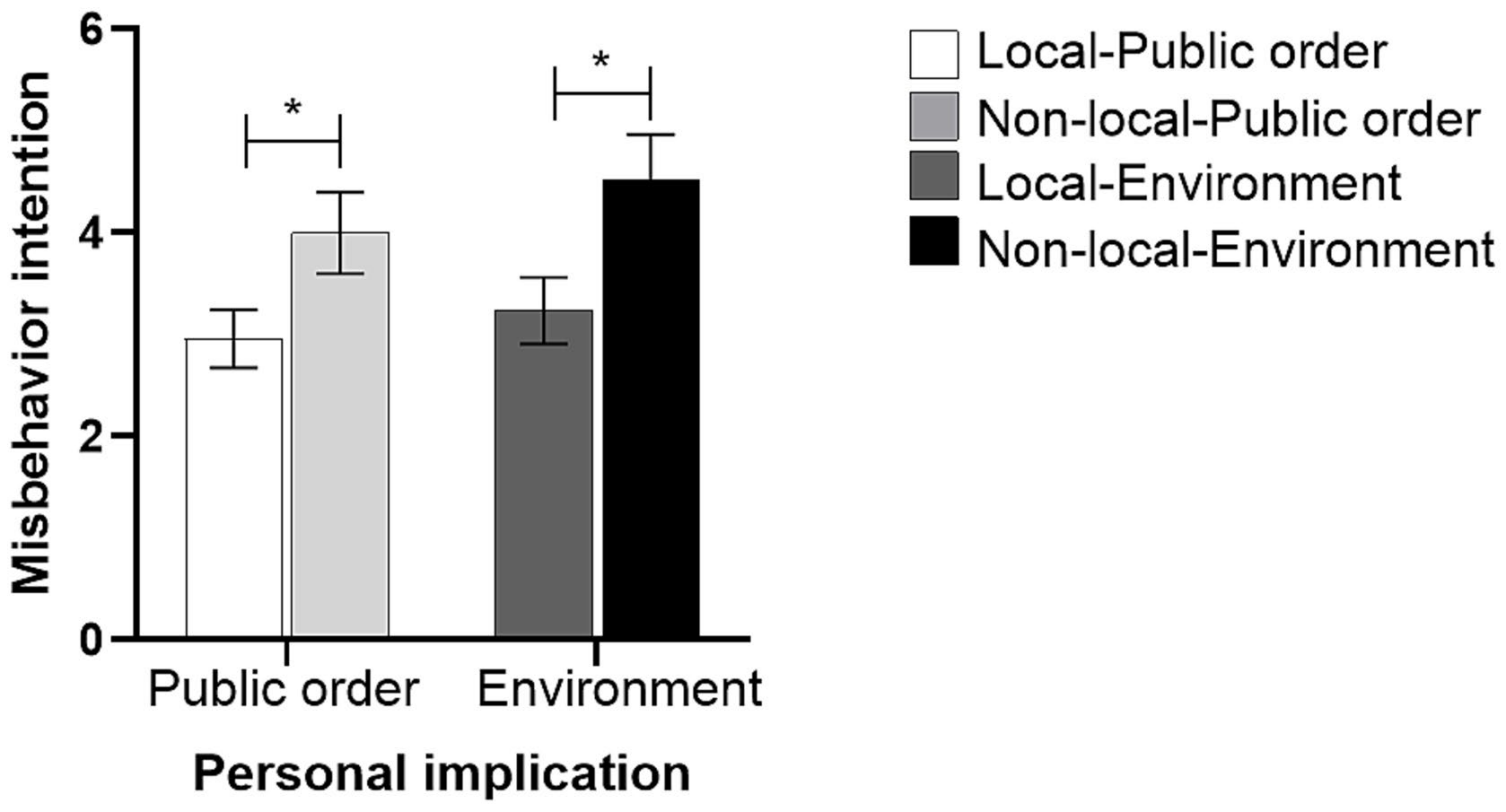
Misbehavior intention of public order and environment while endorsed by local vs. non-local. **p < 0.01. Error bars represent ±1 SE.

Further analysis of the simple effects revealed that, for public order misbehavior, participants in local context was associated with a lower level of misbehavior intention (M_local_ = 2.958, SE = 0.284, M_non-local_ = 3.996, SE = 0.399; *F* (1,40) = 3.128, *p <* 0.05). For environmental misbehavior, participants in non-local context was associated with a higher level of misbehavior intention (M_local_ = 3.235, SE = 0.327, M_non-local_ = 4.517, SE = 0.449; *F* (1,40) = 3.110, *p <* 0.05) (Figure 5).

1. *Response time*. A repeated measures ANOVA with a 2 (psychological distance) × 2 (personal implication) design revealed that the main effect of psychological distance on response time was significant [*F* (1, 40) = 9.774, *p <* 0.01, $\eta_{p}^{2}\;$ = 0.205]. The personal implication did not have a significant main effect [*F* (1, 37) = 1.916, *p* = 0.174, $\eta_{p}^{2}\;$ = 0.048]. However, the interaction effect was found to be significant [*F* (1, 37) = 5.574, *p <* 0.05, $\eta_{p}^{2}\;$ = 0.128].

**Figure 6:**
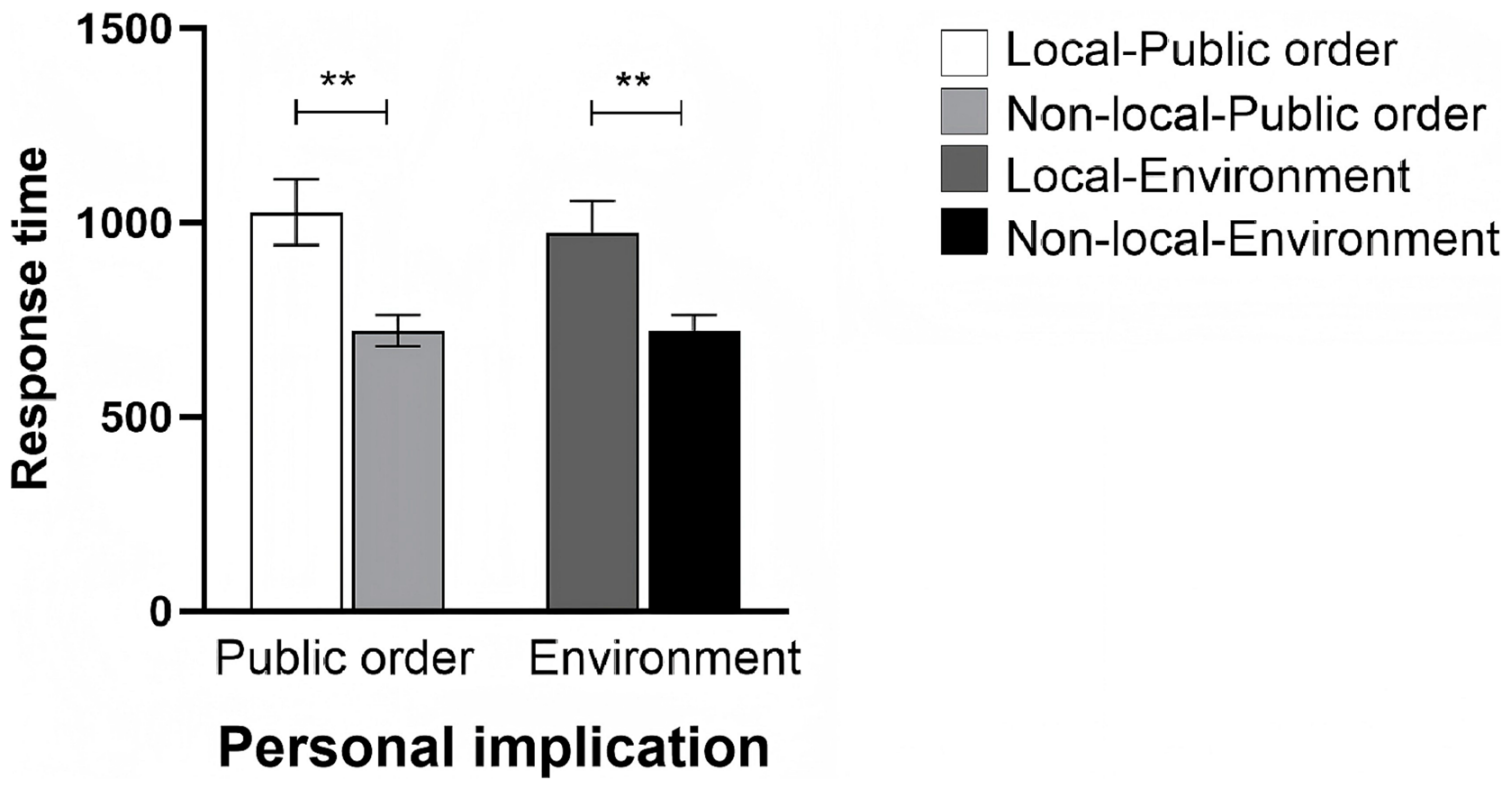
Response time of public order misbehavior and environmental misbehavior while endorsed by local vs. non-local. **p < 0.01. Error bars represent ±1 SE.

Further analysis of the simple effects revealed that, for public order misbehavior, participants in local context led to higher response time (M_local_ = 1,027 ms, SE = 369, M_non-local_ = 720 ms, SE = 172, *F* (1, 40) = 11.668, *p <* 0.01, $\eta_{p}^{2}\;$ = 0.235); for environmental misbehavior, participants in non-local context led to lower response time (M_local_ = 974 ms, SE = 351, M_non-local_ = 734 ms, SE = 182; *F* (1, 40) = 7.558, *p <* 0.01, $\eta_{p}^{2}\;$ = 0.166; see Figure 6).

#### 5.3.2 ERP results

N200: The three-way repeated measures ANOVA with a 2 (psychological distance) × 2 (personal implication) × 6 (electrodes: F3, FZ, F4, FC3, FCZ, FC4) design for the N200 showed that the main effect of psychological distance [*F* (1,40) = 22.062, *p <* 0.001, $\eta_{p}^{2}\;$ = 0.367] is significant. The main effect of personal implication [*F* (1,40) = 9.545, *p <* 0.01, $\eta_{p}^{2}\;$ = 0.201] reached statistical significance. The main effect of electrodes is significant [*F* (1,40) = 22.646, *p <* 0.001, $\eta_{p}^{2}\;$ = 0.345].

**Figure 7:**
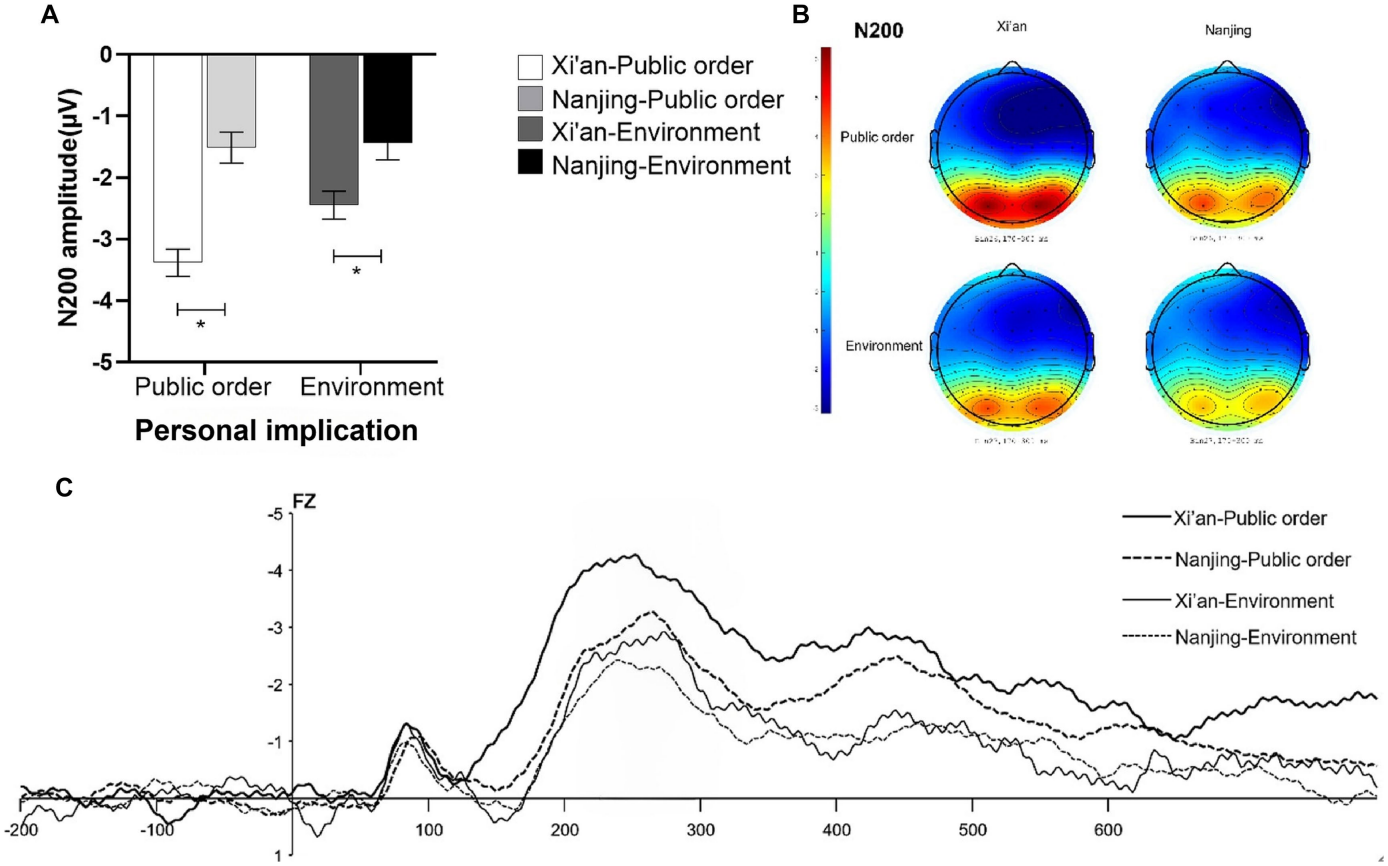
N200 results. **(A)** ERP waveforms at channel mean (time window: 170–300 ms). **(B)** Topographic maps of average N200. **(C)** Bar graph of average N200. *p < 0.05. Error bars represent ±1 SE.

The interaction effect between psychological distance and personal implication was significant [*F* (1,40) = 6.681, *p* = 0.014, $\eta_{p}^{2}\;$ = 0.150]. Simple effect analysis further showed that, in public order misbehavior, the psychological distance evoked by local and non-local contexts differed significantly [M_local_ = −3.380 μV, SE = 0.222, M_non-local_ = −1.512 μV, SE = 0.252; *F* (1,40) = 30.975, *p <* 0.001], participants in local context elicited a larger N200 amplitudes, supporting H4a. In environmental misbehavior, the psychological distance evoked by local and non-local contexts did differ significantly [M_lcoal_ = −2.445 μV, SE = 0.228, M_non-local_ = −1.429 μV, SE = 0.280; *F* (1,40) = 7.931, *p <* 0.01], as shown in Figure 7. Therefore, H4b was supported.

P300: The three-way, 2 (psychological distance) × 2 (personal implication) × 6 (electrodes: P3, Pz, P4, PO3, POz, PO4) ANOVA for the P300 showed that the main effect of psychological distance was significant [*F* (1,40) = 8.011, *p <* 0.01, $\eta_{p}^{2}\;$ = 0.174], and the main effect of the personal implication was significant [*F* (1,40) = 13.549, *p <* 0.001, $\eta_{p}^{2}\;$ = 0.263]. The interaction between personal implication and psychological distance was significant [*F* (1,40) = 4.233, *p <* 0.05, $\eta_{p}^{2}\;$ = 0.100]. Therefore, H5 was supported.

**Figure 8:**
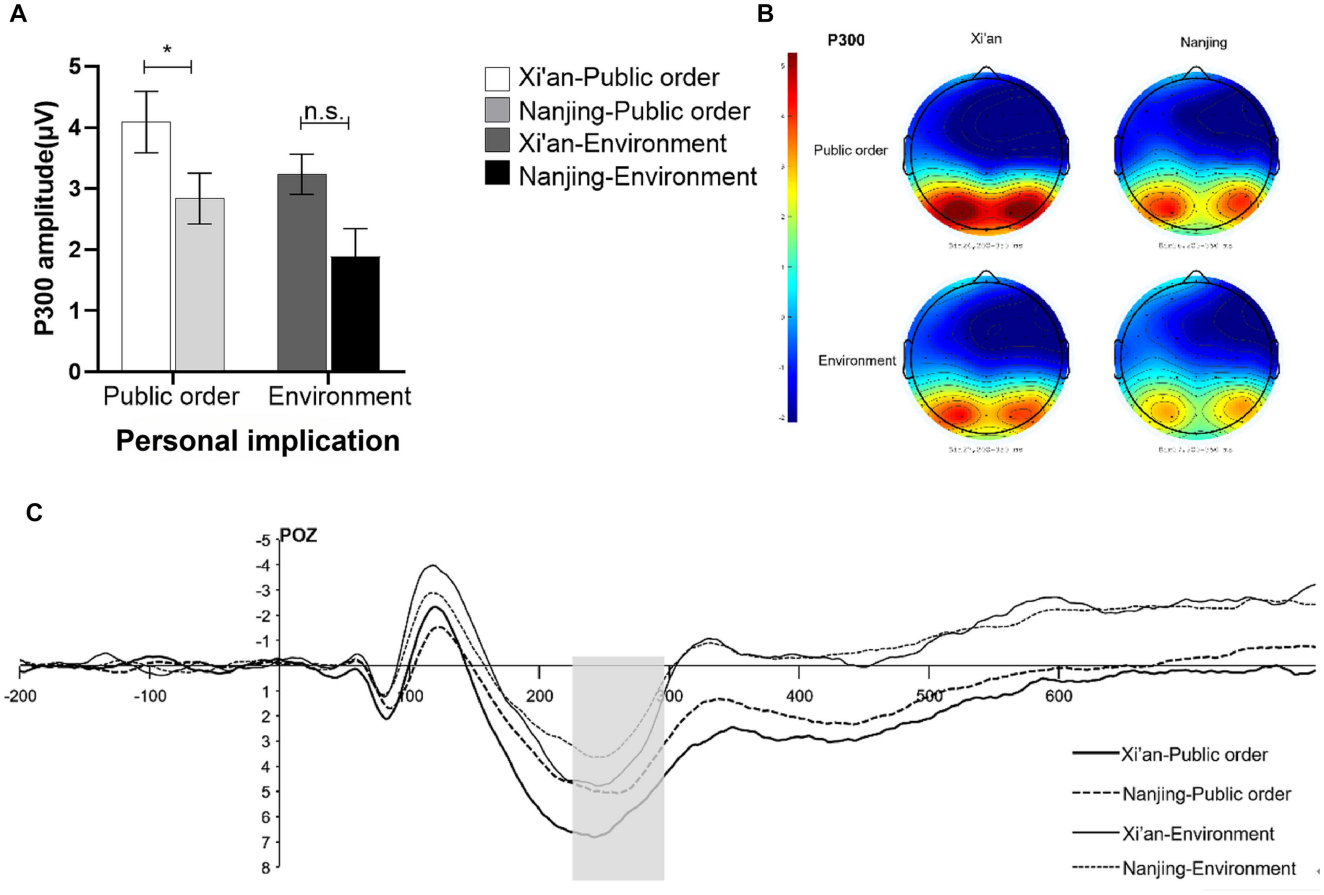
P300 results. **(A)** ERP waveforms at channel mean (time window: 250–380 ms). **(B)** Topographic maps of average P300. **(C)** Bar graph of average P300. *p < 0.05. Error bars represent ±1 SE.

Simple effect analysis further showed that, in public order misbehavior, local elicited a larger P300 [M_local_ = 4.070 μV, SE = 0.528, M_non-local_ = 2.915 μV, SE = 0.402; *F* (1,40) = 5.692, *p <* 0.05\. Thus, H5a was supported. For in environmental misbehavior, the local elicited a larger P300 amplitudes [M_local_ = 3.091 μV, SE = 0.479, M_non-local_ = 2.078 μV, SE = 0.299; *F* (1,40) = 3.689, *p* = 0.062], as shown in Figure 8. Therefore, H5b was not supported.

**Figure 9:**
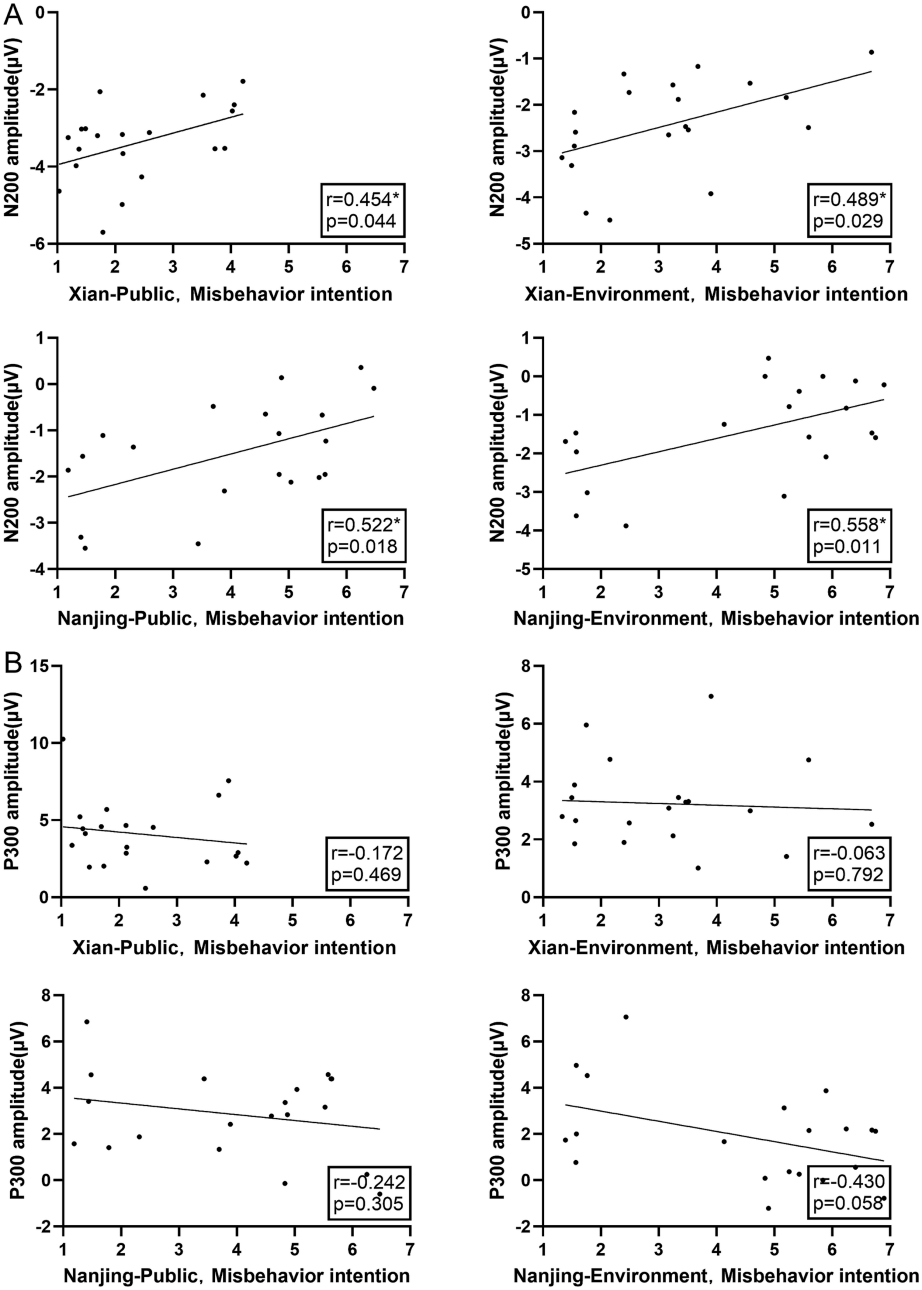
Scatter plots and Pearson correlation coefficient analysis results. **(A)** Misbehavior intention and N200 amplitude. **(B)** Misbehavior intention and P300 amplitude, *p < 0.05.

Correlation analysis: Since the N200 typically peaks in the frontal and frontal-central scalp regions of the scalp (Folstein and Van Petten, 2008), mean value in the frontal and frontal-central areas was selected for correlation analysis. Pearson correlation analysis revealed significant relationship between misbehavior intention and N200 amplitude at mean amplitude between two fixed latencies (see Figure 9A).

Similarly, as the P300 generally peaks in the parietal and parieto-occipital regions (Pozharliev et al., 2015), electrode mean value in the parietal and parieto-occipital regions was selected for P300 correlation analysis. Pearson correlation showed no significant relationship between misbehavior intention and P300 amplitude (see Figure 9B).

## 6 Discussion

With the rapid expansion of participation in sports tourism, understanding and governing tourists’ misbehavior has become a central concern for destination government. However, there remains a lack of systematic explanation for why misbehavior is more prevalent in tourism contexts. To address this research question, the study examines the mechanisms through which psychological distance (local vs. non-local) influences misbehavior intention. Specially, the study introduces informal social control as a key mediator, clarifying how psychological distance shapes perceptions of potential normative responses from bystanders, thereby influencing their misbehavior intention. At the same time, personal implication is incorporated as a critical moderator in the relationship between psychological distance and informal social control, revealing the boundary conditions under which psychological distance affects misbehavior intention. In doing so, this study offers a more nuanced and mechanism-driven explanation of misbehavior in sports tourism context.

Firstly, this study establishes psychological distance as a fundamental driver of misbehavior intention. Specifically, individuals exhibit significantly lower misbehavior intention in local context than those in non-local context, thereby supporting H1. The finding supports the notion that proximity enhances the salience of immediate consequences and social norms, as suggested by construal level theory (Meng et al., 2026; Trope and Liberman, 2010; Bar-Anan et al., 2006). From a moral decision-making perspective, psychological closeness increases self-relevance and embeds behavior within individuals’ social identity and interpersonal context, thereby strengthening perceived responsibility (Sun et al., 2024). In contrast, psychologically distant attenuates perceived responsibility and weaken normative constraints, increasing tolerance for norm violations. The findings are consistent with prior research indicating that increased psychological distance facilitates moral disengagement and reduces adherence to social norms (Jones and Kavanagh, 1996; Wan et al., 2021; Sun et al., 2022). In tourism contexts, similar patterns have been described as a “license to misbehavior,” whereby individuals experience reduced constraint from social norms and a sense of normative unfamiliarity, which may facilitate the subconscious expression of aggressive impulses when leaving their usual place of residence (Uriely et al., 2011; Wan et al., 2021). While prior research has emphasized misbehavior in distant or anonymous settings, the majority of these arguments are conceptual in nature without stringent empirical testing (Sun et al., 2024; Sun et al., 2022). Responding to Uriely, Ram (Uriely et al., 2011) critique that prior accounts of tourist misbehavior are partially overemphasize situational differences while neglecting individuals’ internal psychological processes, the study provides robust empirical evidence that such effects are fundamentally driven by psychological distance, thereby enriching and extending the theoretical framework of misbehavior research. Importantly, this suggests that interventions aimed at reducing perceived distance-such as strengthening social identity or fostering a sense of belonging-may serve as effective strategies for mitigating misbehavior intention.

Secondly, the results clarify the mediating role of informal social control in linking psychological distance to misbehavior intention. In local (vs non-local) context, individuals perceive stronger informal social control, inhibiting misbehavior intention. Thus, H2 is supported. This mechanism is consistent with social control theory (Chekroun, 2008; Brauer and Chekroun, 2005; Janowitz, 1975), which emphasizes the role of perceived social monitoring and potential sanctions in maintaining normative compliance. In line with prior research, perceived social presence and observability have been shown to inhibit misbehavior by increasing reputational concerns and anticipated social costs (Sun et al., 2024; Wan et al., 2021; Brauer and Chekroun, 2005). This study extends the literature by situating informal social control within the interactive and highly participatory context of sports tourism. While existing research has demonstrated the predictive validity of informal social control in fostering normative compliance across multiple social domains-including neighborhoods, families, and peer networks-through mechanisms such as social disapproval, exclusion, and norm internalization (Wan et al., 2021; Chekroun, 2008; Wickes et al., 2017), empirical evidence in transient and highly interactive tourism settings remains relatively limited. Using rigorous experimental designs, the study provides robust empirical evidence supporting these effects and confirms the partial mediating role of informal social control. Collectively, these findings highlight the critical importance of activating informal social control mechanisms in the governance of sports tourism destinations. Importantly, this study demonstrates that informal social control remains operative even in tourism contexts, thereby challenging the prevailing assumption that such mechanisms are weakened under conditions of anonymity or transience (Sun et al., 2024; Wan et al., 2021). In highly interactive and participatory sports tourism, perceived psychological closeness can activate informal control mechanisms despite objective distance, highlighting the importance of situated cognition in norm enforcement progress.

Thirdly, this study identifies significant heterogeneity in the psychological distance-misbehavior mechanism across informal social control, with personal implication emerges as a key boundary condition. Notably, the mediating effect is weaker for public order misbehavior, which involves a higher level of personal implication, as reflected by a smaller indirect effect coefficient. H3a is supported. This pattern is consistent with the broader literature on self-relevance (Zhan et al., 2020; Liberman and Chaiken, 1996) and personal implication (Wan et al., 2021; Chekroun, 2008), which demonstrates that when individuals perceive an event as directly or indirectly affecting their own interests, identity, or experience, they exhibit heightened attentional engagement, stronger normative sensitivity, and greater propensity to act in accordance with perceived social expectations. From a mechanism perspective, public order misbehavior (e.g., queue jumping, pushing, interpersonal disputes) typically involves direct interpersonal interaction and immediate social feedback, thereby increasing perceived self-relevance and activating stronger social evaluative concerns. This heightened personal implication amplifies normative salience and strengthens informal social control processes, as individuals are more likely to anticipate direct social consequences such as disapproval, confrontation, or reputational loss. Consistent with Brauer and Chekroun (2005), perceived personal implication enhances bystander intervention, exerting stronger predictive power than perceived rule violation severity alone. In contrast, environmental misbehavior (e.g., littering, facility damage) is more diffuse and less observable, resulting in weaker norm activation and reduced social constraint. Wan et al. (2021) show in tourism contexts that greater psychological distance reduces anticipated informal sanctions, thereby increasing the likelihood of misbehavior. This finding both aligns with and extends prior research. While earlier studies have emphasized differences in perceived severity, consequences, and interpersonal impact (Sun et al., 2024; Brauer and Chekroun, 2005). The study shifts the focus toward subjective personal implication as a unifying explanatory mechanism, integrating it into a psychological distance-informal social control framework. This refinement clarifies the boundary conditions under which social control mechanisms are activated and suggests that normative regulation in sports tourism is not determined solely by objective behavioral characteristics, but is critically contingent on perceived self-relevance. This study provides a more precise explanation of when and why informal social control is effective.

Fourthly, by incorporating EEG measures, this study offers novel insight into the temporal neural dynamics underlying misbehavior decision-making. Few studies have integrated online behavioral experiments with neurophysiological measures to capture the temporal dynamics underlying such moral decision-making processes. To address this gap, we conducted an ERP experiment (Study 3) that simultaneously manipulated psychological distance (local vs. non-local) and personal implication (public order misbehavior vs. environmental misbehavior). This design enabled us to capture both behavioral outcomes and the underlying cognitive-resource dynamics, thereby linking observable behavioral responses to their neural temporal processes. Specifically, behavioral result showed that non-local contexts elicited higher misbehavior intention than local contexts, and this effect varied across different levels of personal implication. The ERP results revealed that local context elicits longer decision times and larger N200 and P300 amplitudes, indicating greater cognitive control and attentional resource allocation. At the early cognitive processing stage, local contexts elicited larger N200 amplitudes, indicating stronger cognitive control, whereas non-local contexts showed smaller N200 amplitudes. The larger N200 amplitudes observed in local contexts may reflect enhanced activation of the ACC-prefrontal control network. Greater self-relevance in local situations increases sensitivity to norm violations, thereby strengthening early conflict monitoring and cognitive control processes. This pattern is consistent with prior studies showing that, in moral decision-making, compared with strangers (greater social distance), individuals spend more time, experience stronger feelings of aversion, and elicit larger N200 amplitudes related to cognitive control when dilemmas involve friends (closer social distance) (Zhan et al., 2020; Fan et al., 2025). Similarly, results align with Magee and Smith (2013), who found that psychological distance can reduce the intensity of negative feelings. This effect is particularly pronounced in public order misbehavior, where high personal implication amplifies the salience of norm violations, making the negative consequences of misbehavior more salient and urgent to the self, thereby strengthening self-control (Schwartz, 1977). These findings suggest that the interaction between psychological distance and personal implication enhances sensitivity to norm violations, eliciting stronger conflict monitoring and cognitive control during the early stages of information processing. At the later processing stage, local contexts elicited larger P300 amplitudes than non-local contexts, reflecting greater attentional engagement and evaluative processing. Larger P300 amplitudes reflect enhanced engagement of the frontoparietal control network, particularly the dorsolateral prefrontal cortex and parietal regions, which are involved in attentional allocation and evaluative processing. Because local misbehavior is perceived as more self-relevant and consequential, it carries greater motivational significance, prompting individuals to devote more cognitive resources to evaluating norm violations and their potential consequences. Correlation analyses showed that misbehavior intention was positively correlated with N200, or conversely, negatively associated with N200 amplitudes, indicating that stronger cognitive control suppresses misbehavior intention. According to conflict monitoring theory, enhanced N200 reflects successful detection and monitoring of the conflict between misbehavior impulses and social norms, accompanied by the recruitment of executive functions to inhibit inappropriate responses (Folstein and Van Petten, 2008). The results indicate that the enhanced N200 elicited by local public order misbehavior arises from the match between construal level and norm activation, which promotes individual cognitive control. The relationship between misbehavior intention and P300 amplitudes showed a negative but non-significant. Existing evidence indicates that while reduced P300 amplitude is often associated with externalizing or misbehavior, its link to specific deviant or antisocial acts is not consistently significant (Yancey et al., 2013; Bernat et al., 2011). This is because P300 is highly sensitive to stimulus novelty, task relevance, and attentional allocation, yet such neural variations do not necessarily translate into behavioral differences (Iacono et al., 2003). Together, these findings provide convergent neural evidence that psychological closeness strengthens both automatic and controlled processes involved in moral regulation. By linking construal level theory with neurocognitive mechanisms, this study advances a multi-level understanding of misbehavior that integrates cognition and control processes.

### 6.1 Theoretical implications

By distinguishing between public order misbehavior and environmental misbehavior based on the level of personal implication, this study advances the literature on sports tourism misbehavior and deepens theoretical understanding of how psychological distance shapes misbehavior intention, particularly its influence on misbehavior decision-making. Although prior studies have shown that misbehavior is affected by psychological distance (Uriely et al., 2011; Wan et al., 2021; Trope et al., 2007; Vardi and Weitz, 2003), they have not fully elucidated the complex mechanisms through which destination distance operates, nor have they clearly differentiated between types of misbehavior. By introducing this distinction, the study addresses an important gap in the literature. Specifically, this study examines the behavioral and psychological mechanisms through which local versus non-local destinations (as a manipulation of psychological distance) influence sports tourists’ misbehavior intention. While previous research has primarily focused mainly on behavioral outcomes, this study integrates both behavioral and psychological perspectives to explain how psychological distance affects misbehavior. In doing so, it extends the scope of research on misbehavior in sports tourism. This more refined perspective enhances understanding of the interaction between psychological distance and misbehavior, providing new insights for destination image management that go beyond existing findings. This study proposes four key theoretical contributions.

First, this study develops a theoretical framework of psychological distance between tourists and destinations and empirically demonstrates that psychological distance significantly influences intention to misbehavior in sports tourism. Although many studies have introduced the concepts of spatial and social closeness and applied them to general consumers or tourists, systematic research in sports tourism contexts remains limited. Given the distinctions between low psychological distance and high psychological distance in shaping the relationship between destinations and participants (Guo et al., 2019; Liu et al., 2023), this study conceptualizes psychological distance from both physical and psychological perspectives, thereby enriching existing research. Moreover, prior studies on misbehavior have mainly focused on “when to travel” and “with whom to travel” (Su et al., 2022; Manner-Baldeon et al., 2023; Vada et al., 2022; Xu et al., 2018). In contrast, this study extends the scope of travel decision-making to “where to travel” by incorporating spatial and social distance between tourists and destinations. According to the ethical decision-making model proposed by Jones (1991), closeness is a key characteristic of moral issues that influences individual ethical judgments. Although some research has examined the effects of destination closeness and social distance on misbehavior, existing studies have largely concentrated on settings such as hotels, airplanes, and restaurants (Tsaur et al., 2019; Uriely et al., 2011; Xue et al., 2025; Wan et al., 2021; Sun et al., 2022). By comparing different levels of psychological distance at both behavioral and neural levels, this study advances our understanding of the interplay bettween psychological distance and misbehavior intention, contributing new insights beyond prior studies.

Second, this study extends the research on informal social control and clarifies the mechanism through which psychological distance influences misbehavior intention. Although prior studies have examined how psychological distance shapes individual attitudes and behaviors (Guo et al., 2019; Liberman and Chaiken, 1996), as well as how social control affects misbehavior (Bauer and McKercher, 2003; Brauer and Chekroun, 2005), relatively limited attention has been paid to how psychological distance operates through social normative mechanisms to facilitate misbehavior intention. The findings demonstrate that tourists’ perceptions of others’ attention, evaluation, and potential sanctions-that is, informal social control-can be systematically activated by psychological distance and, in turn, significantly influence individuals’ misbehavior intention. While earlier research has confirmed the role of informal social control in promoting norm compliance (Nugier et al., 2009; Wan et al., 2021; Collins and Frey, 1992), this study extends its application by explaining why individual is more likely to engage in misbehavior when participating in sports tourism activities in non-local destinations compared to local ones. In addition, this study employs both behavioral experiments and EEG methodology to rigorously test the proposed hypotheses and provides convergent evidence that perceived informal social control serves as a key underlying mechanism. In doing so, it further enriches the theoretical understanding of how informal social control functions in shaping misbehavior.

Third, this study enriches the sports tourism misbehavior literature by incorporating personal implication as a key contextual factor and moving beyond the homogenized treatment of tourist misbehavior. This distinction addresses the limitation of the “homogenized treatment” of tourist misbehavior in prior research. Although some studies have classified misbehavior according to the degree of disturbance (Loi and Pearce, 2015) or by behavioral consequences (Tsaur et al., 2019; Chekroun, 2008), and others have identified personal implication as an important antecedent of social control responses (Wan et al., 2021; Chekroun, 2008; Brauer and Chekroun, 2005; Janowitz, 1975), these studies provide limited explanation of the causal role of personal implication (Brauer and Chekroun, 2005). The study demonstrates that personal implication not only moderates the relationship between psychological distance and perceived informal social control, but also conditions the indirect effect of psychological distance on misbehavior intention through informal social control. Specifically, the negative impact of psychological distance on informal social control is attenuated in high personal implication contexts, where norm violations are perceived as more self-relevant and consequential, whereas the indirect effect of psychological distance on misbehavior intention becomes stronger in low personal implication contexts. By identifying both the moderating and moderated mediation roles of personal implication, this study provides a more nuanced understanding of the heterogeneous nature of tourist misbehavior and clarifies the boundary conditions under which psychological distance shapes misbehavior through social normative processes. These findings contribute to a more refined theoretical framework for explaining misbehavior in sports tourism settings.

Fourth, by integrating online behavioral experiments with ERP measures, this study provides objective neurophysiological evidence linking observable behavior to its underlying neural mechanisms. Rather than relying solely on self-reported or inferred psychological processes, the ERP results directly capture the temporal dynamics of cognitive processing. Specifically, the identification of a sequential neural pattern-from early conflict monitoring (N200) to subsequent evaluative processing (P300)-offers objective support for the neural pathways through which psychological distance influences misbehavior intention. This evidence-based approach enhances the robustness and validity of the proposed mechanism and advances a more precise understanding of how psychological distance is instantiated in brain activity to regulate behavioral response.

### 6.2 Managerial implications

This study also offers important practical implications. The findings indicate that destination imagery exerts a differentiated influence] on misbehavior intentions. Specifically, the tendency toward public order misbehavior is lower in local destinations, whereas the tendency toward environmental misbehavior is higher in non-local destinations. Perceived informal social control plays a key role in aligning psychological distance with distinct types of misbehavior, which is critical for risk perception and for reducing misbehavior intention.

Accordingly, sports tourism destinations can strengthen tourists’ perception of informal social control through more effective communication strategies and on-site management measures. For example, destinations can enhance reminders regarding the impact of misbehavior on others and increase awareness of the potential consequences and costs of such behavior through clear prompts and signage, thereby discouraging misbehavior. In addition, the ERP findings provide neurophysiological evidence supporting these conclusions, highlighting the importance of matching misbehavior type with destination distance to activate cognitive control process and reduce misbehavior intention. Based on these insights, destination managers and related enterprises can develop targeted strategies to enhance cognitive control and emotional engagement, ultimately improving the governance of tourist misbehavior.

For public order misbehavior characterized by high levels of personal implication (such as standing up to block the view during event, queue-jumping, etc.), sports tourism destinations should prioritize strengthening perceived psychological proximity and informal social control to inhibit the misbehavior intention. Specifically, destination managers can foster a shared identity among tourists, local communities, and other participants by emphasizing narratives such as “this is our home venue” and “civilized viewing ensure a better experience for everyone,” thereby enhancing individuals’ emotional investment and normative sensitivity. In practice, these strategies can be implemented through live broadcasts, on-site digital displays, and timely reminders from volunteers, which highlight the immediate social impact of normal violations on “those around you.” This approach helps activate individuals’ sense of moral responsibility and cognitive control. The effectiveness of these strategies is supported by social identity theory and research on deindividuation research, which demonstrates that stronger group identification and increased social visibility significantly reduced tendencies toward public order misbehavior (Wickes et al., 2017; Pate and Hamilton, 1992).

In contrast, environmental misbehavior with a low level of personal implication (such as taking shortcuts across lawns after events or littering) is more easily rationalized, as its consequences are delayed and the affected parties are less identifiable. In this context, sports tourism destinations should focus on strengthening individuals’ perceived connection and responsibility toward public spaces and the environment, rather than emphasizing whether the behavior is right or wrong. Managers can translate abstract environmental damage into concrete and observable outcomes, for instance through visual displays illustrating the costs of turf restoration, scenarios of ecological damage, or the negative effects on the experience of future events. At the same time, tourists can be guided to view environmental resources as “shared public assets,” thereby strengthening responsibility attribution instead of relying solely on moral appeals. This approach is consistent with behavioral nudging theory and principles of embodied cognition, and it can effectively enhance environmental responsibility in low-involvement contexts by increasing the salience of behavioral consequences and strengthening emotional engagement (Thøgersen, 2021).

### 6.3 Limitations and future studies

Although this study has made progress in explaining the interaction between psychological distance and personal implication, several limitations remain and should be addressed in future research. First, the contextual specificity of sports tourism is not fully captured. Future research is encouraged to incorporate more context-relevant variables-such as sport involvement, team identification, and emotional factors to examine diverse sports tourism settings and cultural contexts in order to enhance the external validity and contextual relevance of the findings. Moreover, although the experimental scenarios were developed based on real-world sports tourism contexts, the study was conducted in a laboratory setting, which may limit ecological validity. Participants’ responses under controlled conditions may not fully reflect actual behavior in natural environments. Future research could enhance external validity by adopting more immersive designs, such as field experiments or context-rich simulations.

Second, regarding the classification of personal implication, although this study distinguishes misbehavior behavior into public order misbehavior and environmental misbehavior based on personal implication-moving beyond the traditional view of misbehavior as a single category-this classification remains somewhat general. Misbehaviors may vary across different types of sports tourism activities (such as Active Sport Tourism, Event Sport Tourism, Nostalgia Sport Tourism, etc.) in terms of social consequences, group norms. Future research could further refine the classification of misbehavior by considering specific types of sports tourism, thereby enhancing the external validity and explanatory power of the findings in complex, real-world contexts.

Third, the sample in this study was mainly from the Chinese population, which may limit the cultural universality of the findings. For example, collectivism, high context, and high power distance societies represented by China have systematic differences in dependence on informal social control, emphasis on “relationship” and “face,” and definition of the boundaries between public order and the private sphere compared with Western individualistic and low-context societies (Gelfand et al., 2011). Specifically, in collectivist cultures, individuals may be more inclined to fulfill their moral responsibilities by maintaining social harmony, and tolerance for misbehavior may also be context-specific due to the differential pattern of “internal and external” (Markus and Kitayama, 1991). Future research should explore whether these observed patterns are universal across different cultural contexts, regional settings, and individual differences.

Finally, in EEG study, “stimulation/task repetition” involves repetition suppression, which often leads to systematic changes in ERP amplitudes such as N1/N140/N170, N2, P2, P3/LPP, etc., covering auditory, visual, emotional and decision-making scenarios, etc. However, prior research indicates that such effects are typically minimal when repetitions are infrequent and widely spaced (Henson et al., 2004), and stimulus repetition is commonly used to enhance signal-to-noise ratio without compromising validity. Nevertheless, the potential influence of habituation cannot be entirely ruled out, and future studies should adopt designs that more directly control to further ensure the robustness of the findings.

## Data Availability

The raw data supporting the conclusions of this article will be made available by the authors, without undue reservation.
