# Supplementary material for: Responses to sports tourist misbehavior: the influence of psychological distance, informal social control and personal implication-behavioral and EEG evidence
Source: Front Psychol. 2026 Jul 13;17:1789417. doi: 10.3389/fpsyg.2026.1789417 (PMC13402421; doi:10.3389/fpsyg.2026.1789417)
Supplement: Supplementary file 1 [file Data_sheet_1.pdf]

Stimulus materials samples (Study 3)

Public order misbehavior

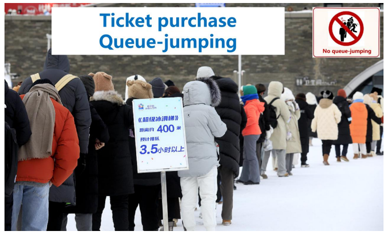

Queue jumping

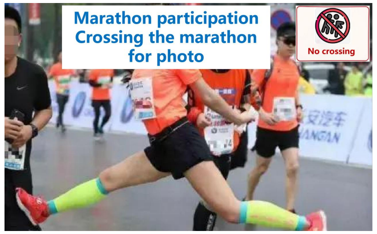

Crossing the track for photo-taking

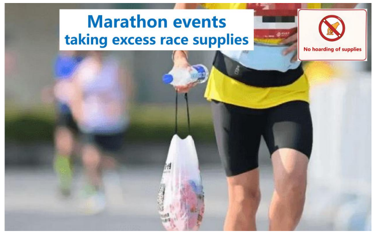

Unauthorized packing of event

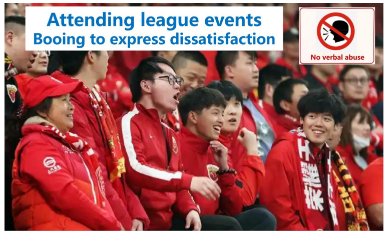

Booing to expressing dissatisfaction

Environmental misbehavior

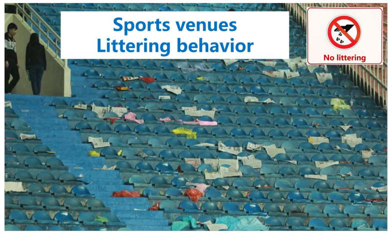

Littering

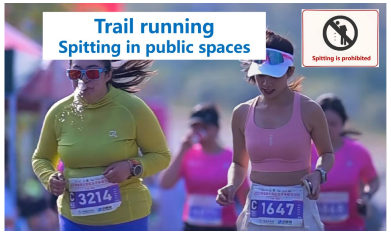

Spitting in public spaces

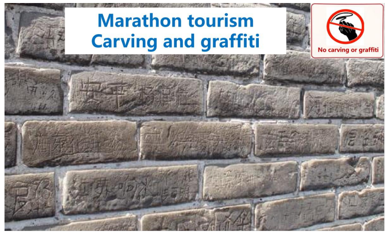

Graffiti

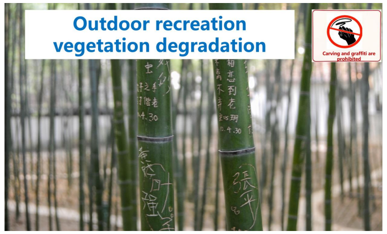

Vegetation destruction

The misbehavior subcategories were operationalized as ecologically valid behavioral scenarios embedded in sports tourism contexts. Specifically, public order misbehavior included queue jumping, crossing the track for photo-taking, unauthorized packing of event supplies, and booing to expressing dissatisfaction. Environmental misbehavior comprised littering, spitting in public spaces, graffiti, and vegetation destruction. All stimulus materials were developed based on realistic sports tourism

scenarios and were carefully matched across subcategories in terms of visual complexity, background setting, actor presence, and scene salience to minimize potential confounding effects.
